# Ornamental plants as vectors of pesticide exposure and potential threat to biodiversity and human health

**DOI:** 10.1007/s11356-024-34363-x

**Published:** 2024-07-24

**Authors:** Cecily Chwoyka, Dominik Linhard, Thomas Durstberger, Johann G. Zaller

**Affiliations:** 1 Department of Integrative Biology and Biodiversity Research, Institute of Zoology, BOKU University, 1180 Vienna, Austria; 2 Umweltforschungsinstitut & Umweltorganisation Global 2000 (Friends of the Earth Austria), Neustiftgasse 36, 1070 Vienna, Austria

**Keywords:** Pesticide residue, Pot plant, Cut flower, Toxic load, Human toxicology, Contamination

## Abstract

The production of conventional ornamental plants is pesticide-intensive. We investigated whether pesticide active ingredients (AIs) are still present in ornamentals at the time of purchase and assessed their potential ecotoxicity to non-target organisms. We purchased 1000 pot plants and 237 cut flowers of different species from garden centers in Austria and Germany between 2011 and 2021 and analyzed them for up to 646 AIs. Ecotoxicological risks of AIs were assessed by calculating toxic loads for honeybees (*Apis mellifera*), earthworms (*Eisenia fetida*), birds (*Passer domesticus*), and mammals (*Rattus norvegicus*) based on the LD_50_ values of the detected AIs. Human health risks of AIs were assessed on the basis of the hazard statements of the Globally Harmonized System. Over the years, a total of 202 AIs were detected in pot plants and 128 AIs in cut flowers. Pesticide residues were found in 94% of pot plants and 97% of cut flowers, with cut flowers containing about twice as many AIs (11.0 ± 6.2 AIs) as pot plants (5.8 ± 4.0 AIs). Fungicides and insecticides were found most frequently. The ecotoxicity assessment showed that 47% of the AIs in pot plants and 63% of the AIs in cut flowers were moderately toxic to the considered non-target organisms. AIs found were mainly toxic to honeybees; their toxicity to earthworms, birds, and mammals was about 105 times lower. Remarkably, 39% of the plants labeled as “bee-friendly” contained AIs that were toxic to bees. More than 40% of pot plants and 72% of cut flowers contained AIs classified as harmful to human health. These results suggest that ornamental plants are vectors for potential pesticide exposure of consumers and non-target organisms in home gardens.

**Supplementary Information:**

The online version contains supplementary material available at 10.1007/s11356-024-34363-x.

## Introduction

The global use of pesticides has increased in recent decades (Persson et al. 2022; Zaller 2020), which has led to a decline in biodiversity and negative impacts on human health (Brühl & Zaller 2019; Cech et al. 2023; Egendorf et al. 2020; Gaspari et al. 2020; MacFarlane et al. 2013; Tang et al. 2021). The effects of pesticide exposure have mainly been studied in an agricultural setting (Alengebawy et al. 2021; Linhart et al. 2021; Varghese et al. 2021; Zaller et al. 2023b), while much less attention has been paid to the use and fate of pesticides used in ornamental plant production (Lentola et al. 2017; Toumi et al. 2017). However, ornamental plant production is one of the most pesticide-intensive sectors (Zaller 2020) and already shows signs of pesticide overuse, such as multiple resistance of plant pathogens (Fraaije et al. 2021; Takahashi et al. 2021). In addition, the global trade in ornamental plants has increased in recent decades, with the largest demand coming from the European Union (EU), where sales of several billion Euros are generated annually (Pereira et al. 2021). As reported by non-governmental organizations (NGOs) in North America (FoE 2014, 2016) and Europe (Global 2000 2022, Greenpeace 2014) as well as scientific studies (Lentola et al. 2017; Toumi et al. 2017, 2016a), the pesticides used in the cultivation of pot and cut flowers are still present at the time of sale. However, their potential toxicity to non-target organisms, including humans, has hardly been assessed (BfR 2021; Lentola et al. 2017; Pereira et al. 2021; Porseryd et al. 2024).

Hundreds of pesticides are used in ornamental plant production to ensure high esthetic quality, high production rates, and compliance with regulations of importing countries, which require the absence of pests and pathogens (Bethke & Cloyd 2009; Pereira et al. 2021; Toumi et al. 2017). The most commonly used pesticide classes are insecticides, miticides, and fungicides, with organophosphates, carbamates, triazoles, and pyrethroids being the predominant chemical category (Bethke & Cloyd 2009; Pereira et al. 2021; Toumi et al. 2017, 2016a). As a result, ornamental plants imported into the European Union (EU) are contaminated with a wide range of pesticides that could be toxic to non-target organisms, including humans. Exposure of non-target organisms to pesticides on ornamental plants can occur through direct contact with contaminated plant surfaces (dermal exposure), through inhalation of contaminated airborne particles due to evaporation of pesticides (inhalation exposure) or, in the case of edible plants or herbs, through ingestion of contaminated plant material including nectar and pollen (oral exposure) (Cech et al. 2022; Etheredge & Waliczek 2022; Lentola et al. 2017; Oliveira et al. 2021; Tassin de Montaigu & Goulson 2020; Zaller et al. 2022). Studies have shown that pesticides used in cut flowers can easily be absorbed through the skin of florists when they prepare bouquets and handle contaminated flowers; eight pesticide residues and metabolites were detected per urine sample of Belgian florists, which also has potential implications for their health (Toumi et al. 2020). Humans exposed to pesticides may experience weakness, muscle pain, fever, or headaches as well as chronic effects such as neurological and mutagenic damage, prematurity, endocrine disorders, mental health problems, or cancer (Boedeker et al. 2020; Bouvier et al. 2006; Burtscher-Schaden et al. 2022; Gaspari et al. 2020; Toumi et al. 2016a).

Pesticides in ornamental plants can also affect the health of insects that obtain nectar and pollen from them (Lentola et al. 2017). However, not much is known on potential effects on other non-target animal species that may come into contact with pesticide-contaminated ornamental pot plants in the garden or with wilted cut flowers that are composted in the garden. The ecotoxicity of pesticides is evaluated through environmental risk assessments using standardized tests on surrogate animal groups such as honeybees, birds, earthworms, and mammals (Donley 2019; OECD 1984, 1998, 2016). Studies have shown that pesticides can cause disorientation, behavioral changes, paralysis, or even death of honeybees (Cecala & Wilson Rankin 2021; Goulson et al. 2015; Kalaman et al. 2020; Lentola et al. 2017; Tosi et al. 2022). Earthworms have been shown to be affected by pesticides by altering their behavior and reproduction (Fernández-Gómez et al. 2011; Pelosi et al. 2014; van Hoesel et al. 2017; Zaller et al. 2016). Birds are affected by pesticides in their survival, reproduction, mobility, navigation, and population dynamics (Moreau et al. 2022; Tassin de Montaigu & Goulson 2020). Mammals can respond to pesticide exposure with miscarriages, skeletal abnormalities, DNA damage, organ size reduction, and altered activity levels in mammals (Berheim et al. 2019; Klátyik et al. 2023; Royte 2021). In freshwater organisms, the ecotoxicity of pesticides used in ornamental horticulture was much higher than in field crops due to high application rates and their intrinsic toxicity (Yin et al. 2023).

The aim of this study was therefore to (i) analyze the extent of pesticide contamination of ornamental plants purchased in garden stores, (ii) assess the potential toxicity of the detected pesticide residues to a range of non-target organisms based on the LD_50_ values of the active ingredients (AIs) and to human health based on their hazard statements, and (iii) investigate associations between pesticide contamination and plant characteristics (species, country of origin, advertising as pollinator-friendly). The scenario on which our study was based was that pesticide-contaminated ornamental plants are placed as pot plants in the home garden or planted in the flower bed or that wilted cut flowers are composted in the garden. In the garden, pollinating insects foraging for nectar and pollen could come into contact with pesticides in ornamental plants (Botías et al. 2017; David et al. 2016), birds could be exposed by eating contaminated seeds (Eng et al. 2017), and rats and earthworms could feed on contaminated cut flowers in a compost heap (Tariba Lovaković et al. 2017; Wagman et al. 1999). Therefore, we selected the western honeybee (*Apis mellifera*), the compost worm (*Eisenia fetida*), the house sparrow (*Passer domesticus*), and the brown rat (*Rattus norvegicus*) for our assessment of potential pesticide effects because they provide essential functions and services in garden ecosystems and are also used as surrogate species in official environmental risk assessments of pesticides. However, it is important to emphasize that, in reality, only a small proportion of pesticide residues in ornamentals are taken up by these species, and this proportion depends on the persistence of the AIs and many other factors. Furthermore, the effects are different when multiple pesticides are present, and sublethal effects can occur at doses much lower than the LD_50_. Nevertheless, the results of this study can highlight the extent of pesticide contamination of ornamental plants and raise awareness that the use of pesticides on ornamental plants and cut flowers can contribute to the exposure of non-target organisms, including humans, to chemicals of concern.

## Materials and methods

### Pesticide residue data

Ornamental pot plants and cut flowers were purchased between from 22 different garden centers, DIY stores, grocery stores, and/or nurseries in Austria and Germany between 2011 and 2021. The plant samples for the analysis of pesticide AI residues were taken by garden center employees or employees of the Environmental Research Institute Global 2000 according to a harmonized sampling protocol. Due to a data protection agreement, the respective markets cannot be named here.

The selection criteria for sampling of plant species focused preferably on imported plants that were in high demand in the garden centers and on plants that were labeled as “pollinator-friendly.” Over the years, we took between one and 220 pot-samples per year (1000 pot plants in total) and between 2 and 47 cut flower samples per year (237 cut flowers in total; Supplementary Table S1). The pot plants were mainly produced in Europe, while most of the cut flowers came from Tanzania and Kenya. The analyses for pesticide residues in plant samples were carried out by 10 accredited laboratories in Germany and Austria; 95% of the samples were analyzed by three laboratories over the years. The commercial laboratories used the QuEChERS method in accordance with OENORM EN 15662:2018 (OENORM 2018). Subsequently, LC–MS/MS, GC–MS/MS, and GC-ECD multi-methods were performed to determine a maximum of 646 AIs per year and an average 552 ± 53 AIs (mean ± SD). The limits of quantification were between 0.005 and 0.020 mg kg^−1^. Occasionally, additional analyses were performed for dithiocarbamates (DTC), chlormequat, or phosphorous acid. Supplementary Table S2 gives an overview of the AIs and the respective limits of quantification used by the three main laboratories, which performed 93% of all analyses over the years.

The pot plant samples included 302 plant species, 12 unidentified plant species, and 28 samples which were labeled as “mixtures” of different plant species in the original dataset. The most frequently analyzed pot plant species were *Euphorbia pulcherrima* (77 samples), *Lavandula angustifolia* (45 samples), and *Rosa* spp. (29 samples). Cut flower samples comprised 96% (227 samples) *Rosa* spp., *Aralia* spp. (2 samples), *Dianthus* spp. (1 sample), *Gerbera* spp. (1 sample), *Tulipa* spp. (4 samples), and species mixtures (2 samples). Depending on the size of the plants, 3–12 individuals were used as a composite sample. The persons who took the samples were instructed to use nitrile gloves, to change gloves for each new sample and store the individual samples in plastic bags to avoid cross-contamination. As the samples were usually sent to the laboratories within 1–2 days, they were not frozen or otherwise preserved.

### Additional data collection

All detected pesticides were characterized using the Pesticide Properties Database (PPDB) of the University of Hertfordshire from 2022 (Lewis et al. 2016). This included information on the pesticide category, the substance group, the mode of action, various ecotoxicological data, and whether the AI is approved in the EU according to the EC Regulation 1107/2009 status. If several pesticide categories were mentioned for an AI in the PPDB, the first one mentioned was selected.

Due to a lack of information in the PPDB, 7 AIs found in pot plants (perchlorate, chlorate, fentin, nitrate, desmethyl pirimicarb, folpet phthalimide, and captan (sum of captan and tetrahydrophthalimide THPI)) and two AIs found in cut flowers (fm 6–1 and 4-brom-2-chlorphenol) were not included in our analysis. Each of these omitted AIs was detected less than 7 times over the years and thus represented a very small proportion of the total sample. Consequently, the total number of AIs considered for toxicological evaluation was 195 AIs for pot plants and 128 AIs for cut flowers.

In addition, all plant species examined were categorized according to whether they can be kept indoors or outdoors (or both) in a Central European climate and whether they are organically grown, according to the product label. The classification of pollinator friendliness of plants was adopted from the Royal Horticultural Society (RHS 2023). If the plant species was not included in the original dataset, this information was marked as “n/a” (information not available).

### Evaluation of ecotoxicity

**Fig. 1:**
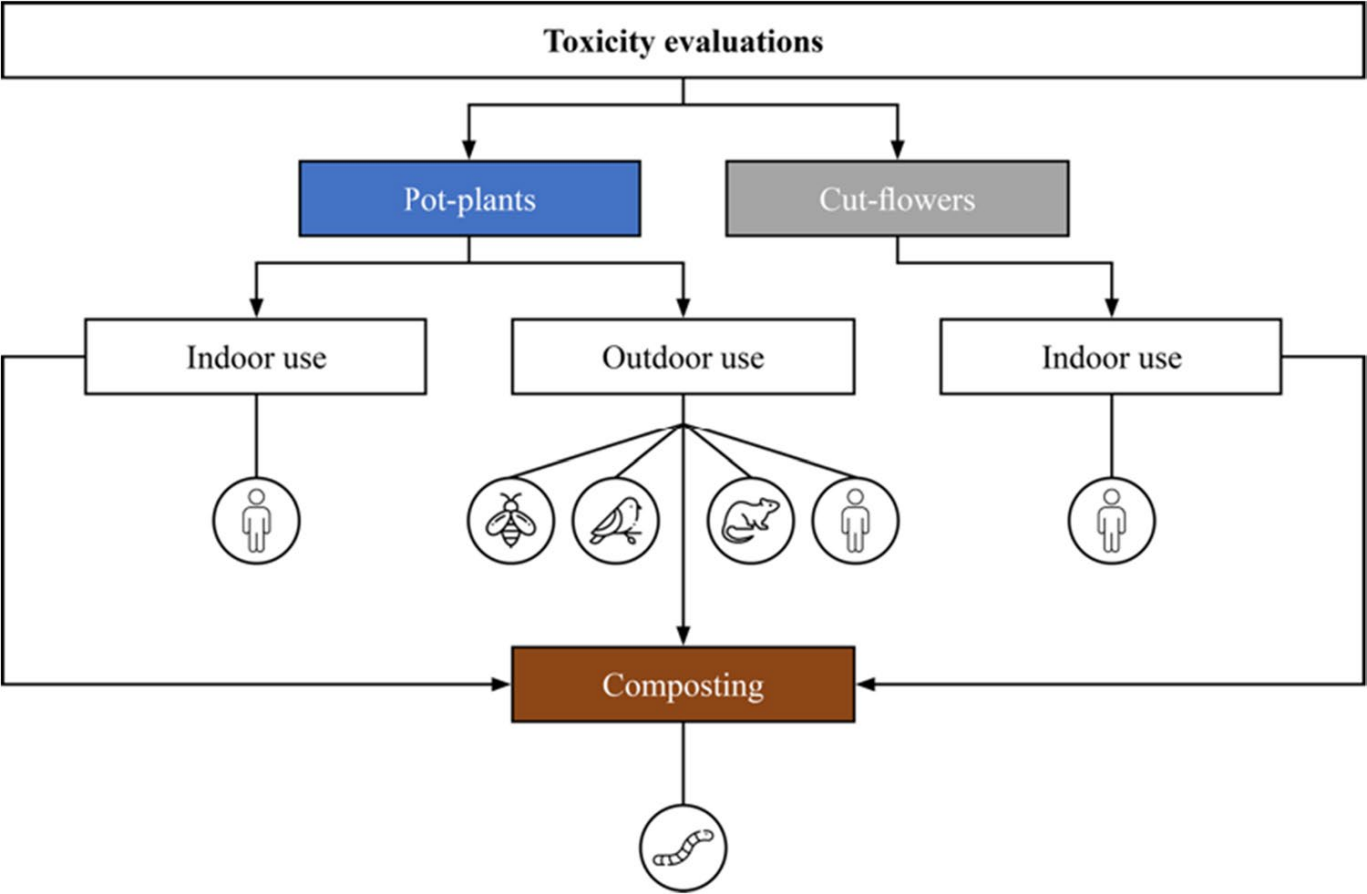
Overview of the scope of toxicity calculations and evaluations for honeybees, earthworms, birds, rats, and humans. The organism symbol under the plant category (indoor or outdoor) indicates which organismic group is accounted for in the analysis of the respective plant species

To assess the toxicity of pesticide residues detected in ornamental plants, surrogate species commonly found in residential gardens were selected: the western honeybee (*Apis mellifera*) as a potential pollinator, visitor, and forager of ornamental plant nectar and pollen planted in gardens; the compost worm (*Eisenia fetida*), which could come in contact with composted cut flowers; the house sparrow (*Passer domesticus*), which could feed on ornamental plant seeds; and the brown rat (*Rattus norvegicus*) as a representative of mammals such as rabbits, which could come into contact with contaminated ornamental plants or eat contaminated earthworms. Other beneficial insects such as parasitic wasps and predatory mites, which are taken into account in ERAs, were not included in this analysis. Depending on the plant characteristics (indoor or outdoor), different toxicity assessments were considered relevant. For outdoor pot plants, toxicity to honeybees, earthworms, birds, and mammals was assessed. Cut flowers, on the other hand, are mainly kept indoors, so it was assumed that only the compost worm comes into contact with potentially hazardous residues when composting the cut flowers in the garden. The human toxicity of the detected AI residues was assessed for all cut flowers and pot plants. Figure 1 shows an overview of the species considered for each plant category.

The overall ecotoxicity of the detected AIs was assessed using the median lethal dose LD_50_ or lethal concentration LC_50_ causing death in 50% of the population studied (Lewis et al. 2016), the no-observed-effect concentration NOEC, the no-observed-effect level NOEL, and the no-observed adverse effect level NOAEL values of the respective substances given in the PPDB (Lewis et al. 2016). Where LD_50_ or LC_50_ values were given with a “greater than” sign (e.g., flonicamid > 2000 mg kg^−1^), the value was considered equal to the number (e.g., 2000 mg kg^−1^). Consequently, toxicity was overestimated in these cases. This procedure is consistent with other studies (Cech et al. 2022; Goulson et al. 2018; Tassin de Montaigu & Goulson 2020). The NOEC, NOEL, and NOAEL values indicate the concentration or level of an AI that causes no observed (adverse) effect and are used to assess the ecotoxicity during chronic exposure of organisms. An interpretation of LD_50_, LC_50_, NOEC, NOEL, and NOAEL values for each organism, presented as toxicity categories, is summarized in Supplementary Table S3.

When assessing the toxicity of pot plants and cut flowers, the sample was categorized according to the most toxic AI detected in case the plant was contaminated with multiple AIs. For example, if fipronil (high acute toxicity to birds) and boscalid (moderate acute toxicity to birds) were detected in the same plant sample, it was categorized as highly toxic to birds.

In addition, the persistence of the pesticide (half-life) provided in PPDB was taken into account (Supplementary Table S4). Persistence represented the DT_50_ value derived from laboratory or field studies, which defines the degradation time required to reduce an initial quantity/concentration by half. For this study, the DT_50_-field value was preferably used for all AIs. If no DT_50_-field value was given, the DT_50_ typical value as a mixture of laboratory and field studies given in PPDB was used. This was the case for 27 pesticides for pot plants and 4 pesticides for cut flowers. If no DT_50_ value was provided in the PPDB, other sources were consulted in the following order: PubChem (https://pubchem.ncbi.nlm.nih.gov), ECHA (https://echa.europa.eu), registration reports, and articles in peer-reviewed journals. This procedure was used for 4 pesticides for pot plants (didecyldimethylammonium chloride (DDAC), diphenylamine, mepiquat, and methoprotryne) and 2 pesticides for cut flowers (DDAC and matrine). No DT_50_ value was found for benthiavalicarb, benzalkonium chloride, diammonium ethylenebis (dithiocarbamate), and ethiofencarb sulfone.

### Toxic load calculations

To evaluate potential ecotoxicological risks of pesticide residues for organisms in residential gardens, toxic load (TL) calculations were carried out. The TL depends on the AI toxicity (LD_50_) and the amount applied and was additionally weighted by the persistence (DT_50_) of the respective AI. It is assumed that a higher persistence means a higher exposure risk for non-target organisms over a longer period of time, which leads to a higher TL. Similar calculations of the toxic loads of AIs have been performed in several studies (Cech et al. 2023, 2022; Goulson et al. 2018; Kudsk et al. 2018; Neumeister 2017). All TLs were calculated using Eq. (1) (Cech et al. 2022):

1 $$Toxic\;load \left(TL\right)=\sum^{{n}_{AI}}\frac{Pesticide\;residue}{{LD}_{50 AI}}* \frac{{DT}_{50 AI}}{ln(2)}$$

Consequently, the TLs were calculated by dividing the AI concentration (mg kg^−1^ plant material) by the LD_50_ value of the respective AI. The units of the LD_50_ value varied by species: μg bee^−1^ for honeybees and mg kg_bw_^−1^ for earthworms, birds, and mammals. Then, the TLs were multiplied by the persistence of the pesticide, resulting in persistence-weighted TLs. Finally, the obtained term was divided by the natural logarithm of 2, which represents the first-order degradation kinetics (Cech et al. 2022). This result is TLs, expressed in LD_50_ days per kg of plant material, which indicate the number of days a given LD_50_ dose is present on a kg of plant material.

Only AIs for which LD_50_/LC_50_ and DT_50_ values were available were considered in the TL calculations: for pot plants, this resulted in 179 AIs for honeybee contact toxicity (16 AIs excluded), 174 AIs for honeybee oral toxicity (21 AIs excluded), and 179 AIs for earthworm toxicity (16 AIs excluded), for birds 184 AIs (11 AIs excluded), and mammals 190 AIs (5 AIs excluded). For cut flowers, 118 AIs for earthworms (8 excluded) and 124 AIs for mammals (2 AIs excluded) were included in the TL calculations. Only pot plants for outdoor use were included in the TL calculations for honeybees, birds, and mammals (Fig. 1). In addition, pot plants that could not be categorized for either indoor or outdoor use due to their nonspecific botanical identification, as well as plants intended for both indoor and outdoor use, were included in these calculations. Any pot plant, whether used for indoor or outdoor use, was included in the TL calculations for earthworms.

For honeybees, LD_50_ values in μg bee^−1^, expressed as worst-case values of 24, 48, and 72 h of exposure, were used. Therefore, to calculate the TLs, the residue levels detected in plant material (μg kg^−1^) were divided by the LD_50_ values. As data were available for oral and contact exposure, two separate TL calculations were performed for honeybees (Cech et al. 2022; DiBartolomeis et al. 2019). These TLs indicate the potential kills due to the amount of the detected AIs in plant material, assuming that all material was consumed by or came into contact with honeybees. It is important to emphasize that this TL calculation is only an attempt to illustrate the potential ecotoxicological impact of pesticide residues and not to estimate actual bee kills, for which we do not know the actual proportion of AI that come into contact with bees. We also recognize that this simple analysis does not account many factors, such as the effects of multiple pesticide residues, chronic exposures, multigenerational impacts, endocrine disruption, and non-monotonic dose–response relationships that will affect actual exposure of non-target organisms.

For earthworms, acute 14-day LC_50 EW_ values were reported in mg kg^−1^ bodyweight by PPDB. To obtain the LD_50 EW_ values (mg EW^−1^), the following Eq. (2) was used (Cech et al. 2022):

2 $${LD}_{50\;EW}=\frac{{LC}_{50\;EW}}{\phi n_{EW\;}per\;kg\;compost\;soil}$$

The average amount of earthworm (*ø n*_EW_) kg^−1^ compost soil was calculated using the following data from previous studies: mean bulk density of compost soil of 537.5 kg m^−3^ (mean of 420 kg m^−3^ and 655 kg m^−3^) (Khater 2015), mean weight of adult *Eisenia fetida* 0.55 g (Schmidt et al. 2023), and mean earthworm loading of 0.298 kg (mean of 0.17 kg, 0.26 kg, 0.34 kg, and 0.42 kg) in compost worm bins of 0.0532 m^2^ (0.56 m × 0.38 m × 0.25 m) (Ndegwa et al. 2000). This resulted in an average of 18.92 earthworms kg^−1^ compost soil.

To assess the potential risk of pesticide residues on ornamental plants to garden birds, the TL for house sparrows (*Passer domesticus*) was calculated instead of the surrogate species used in standard environmental risk assessments (Virginia quail, Japanese quail, mallard, gray partridge, red-legged partridge, and Atlantic canary), as these do not live in gardens in Central Europe. For this purpose, acute oral LD_50_ values, given in mg kg_bw_^−1^, were used for several surrogate species from the PPDB (Lewis et al. 2016). Data available in the PPDB varied among Virginia quail (*Colinus virginianus*), Japanese quail (*Coturnix japonica*), mallard (*Anas platyrhynchos*), gray partridge (*Perdix perdix*), red-legged partridge (*Alectoris rufa*), and Atlantic canary (*Serinus canaria*). Due to lack of data on LD_50_ values of any surrogate bird species, 10 AIs had to be excluded from the calculations. For the TL calculations, LD_50_ values in mg kg_bw_^−1^ were converted in mg bird^−1^ by multiplying the LD_50_ by the average body weight of the respective surrogate bird. For this purpose, an average weight was calculated for each bird species based on values in the literature: *C. virginianus* 0.174 kg (Mohlman et al. 2020; Roseberry & Klimstra 1971), *A. platyrhynchos* 1.057 kg (Brewer et al. 2003; Janiszewski et al. 2018; Kline et al. 2011), *C. japonica* 0.095 kg (Cech et al. 2022), *P. perdix* 0.371 kg (Homberger et al. 2014, Liukkonen‐Anttila et al. 1999, Pis 2008), *S. canaria* 0.022 kg (Harper & Turner 2000), and *A. rufa* 0.415 kg (Lopez-Antia et al. 2013; Millan et al. 2003; Negro et al. 2001). Subsequently, the LD_50_ values in mg per surrogate bird species were extrapolated according to Eq. (3) (Tassin de Montaigu & Goulson 2020) to obtain the LD_50_ value for *P. domesticus*:

3 $$\mathit{log}\left({LD}_{50 2}\right)=\mathit{log}\left({LD}_{50 1}\right)+\left(\mathit{log}\left({W}_{2}\right)-\mathit{log}\left({W}_{1}\right)\right)*1.239$$

LD_50 1_ and W_1_ represent the LD_50_ value and body weight of the surrogate species. LD_50 2_ and W_2_ represent the LD_50_ and body weight of the house sparrow. The average weight for *P. domesticus* was assumed to be 0.028 kg (Fischer et al. 2018, Schafer et al. 1973, Singer & Yom-Tov 1988). The scaling factor of 1.239 was used to improve the extrapolation process (Tassin de Montaigu & Goulson 2020).

The TL for rats (*Rattus norvegicus*) was calculated as a surrogate for terrestrial, herbivorous mammals potentially affected when eating or coming into contact with contaminated pot plants in residential gardens. Acute oral LD_50_ values for mammals (mg kg_bw_^−1^) were reported by PPDB for several surrogate species, including rats and mice (*Mus musculus*) in the case of cut flowers and rats, mice, and rabbits (*Oryctolagus cuniculus*) in the case of pot plants. The LD_50_ values were first multiplied by the average body weight of the respective surrogate species to obtain LD_50_ values in mg per rat, mouse, or rabbit. For this purpose, an average body weight of 0.213 kg was assumed for *R. norvegicus* (Alwan et al. 2021; Isdadiyanto et al. 2020; Islam et al. 2021), 0.022 kg for *M. musculus* (Ademola et al. 2020; Arundina et al. 2020; Islam et al. 2021), and 1.772 kg for *O. cuniculus* (Eladl et al. 2020; Mary Momo et al. 2019; Rehman et al. 2022).

In the following, LD_50_ values (mg per surrogate species) for mice and/or rabbits were extrapolated to obtain LD_50_ values for rats (*Rattus norvegicus)*. The extrapolation was performed according to the following Eq. (4):

4 $$\mathit{log}\left({LD50}_{2}\right)=\mathit{log}\left({LD50}_{1}\right)+\left(\mathit{log}\left({W}_{2}\right)-\mathit{log}\left({W}_{1}\right)\right)*0.94$$

While LD50_1_ and W_1_ indicate the LD_50_ values and body weights of the respective surrogate species (mouse or rabbit), LD50_2_ and W_2_ represent the LD_50_ and body weight of a rat. A scaling factor of 0.94 was applied based on previous studies (Sample & Arenal 1999).

Again, it is important to note that all TL calculations performed here only indicate potential hazards and not actual mortalities. As already mentioned for honeybees, actual amount of intake or contact with pesticide residues determine the real exposure and is not considered in this calculation (Cech et al. 2022; Goulson et al. 2018). As the effects of AIs were analyzed separately, synergistic or cocktail effects, were not taken into account (EFSA et al. 2019; Geissen et al. 2021).

### Evaluation of human toxicology

To evaluate the human health hazards, all AIs were classified according to the hazard statements of the Globally Harmonized System of Classification and Labelling of Chemicals (GHS) as defined by the EU regulation (EC) 1272/2008 (UN 2021). The respective GHS hazard statements for each pesticide were taken from the EU Pesticide Database, PubChem, as well as the PPDB (Supplementary Table S5). Such an approach to human toxicology assessment has been performed in previous studies (Burtscher-Schaden et al. 2022; Cech et al. 2023; Silva et al. 2022; Zaller et al. 2022, 2023a). No human health hazard data were found for benzalkonium chloride, diammonium ethylenebis (dithiocarbamate), and tetrahydrophthalimide in pot plants and for benzalkonium chloride in cut flowers, so they were excluded from this analysis. This resulted in 192 AIs for pot plants and 125 AIs for cut flowers that were included in the analysis.

In addition, all AIs were screened for their effects on the endocrine system according to the PPDB. Pesticides listed as either potentially or confirmed endocrine disruptors were categorized as “endocrine disrupting.” If a plant sample was contaminated with at least one potentially cancerogenic AI, it was categorized as containing a cancerogenic AI. AIs without a hazard statement were classified as not harmful to human health.

## Results

### Pesticides detected

A total of 202 different AIs were detected in ornamental pot plants and 128 AIs in cut flowers (Table 1). However, sufficient information for ecotoxicological analysis could only be obtained for 195 and 126 of these AIs, respectively. Of all AIs detected in pot plants, 34% (69 samples) were insecticides, 14% (28) herbicides, and 36% (71) fungicides (Supplementary Fig. S1). Of the AIs detected in cut flowers, 44% (55) were insecticides, 2% (3) herbicides, and 48% (61) fungicides. Overall, the top-5 AIs with the highest concentrations in pot plants included chlormequat (750 mg kg^−1^), 1-naphthylacetamide (649 mg kg^−1^), folpet (160 mg kg^−1^), fenhexamid (157 mg kg^−1^), and paclobutrazol (155 mg kg^−1^) (Supplementary Table S5). For cut flowers, the top-5 AIs with the highest concentrations were clofentezine (190 mg kg^−1^), propamocarb (94 mg kg^−1^), iprodione (76 mg kg^−1^), BAC (53 mg kg^−1^), and dodemorph  (50 mg kg^−1^) (Supplementary Table S6).

**Table 1:** Overview of the number and characteristics of AIs detected on pot plants and cut flowers. Means ± SD

Number of…	Pot plants	Cut flowers
Samples analyzed	1000	237
AIs detected/ecotoxicologically evaluated	202/195	128/126
AIs detected per plant on average	5.76 ± 4.03	10.97 ± 6.2
AIs detected per organic plant on average	2.3 ± 2.4	0
AIs not approved in the EU as of 2022	72	53
Samples with > 1 AI detected	849	233
Samples without contamination/proportion	63/6.3%	1/0.4%

According to the status of EC Regulation 1107/2009, 35.6% of AIs detected in pot plants and 41.4% of AIs detected in cut flowers are not approved in the EU in 2022 (Table 1). While pot plants were contaminated with an average of 5.76 ± 4.03 AIs (mean ± SD), this number was almost twice as high for cut flowers (10.97 ± 6.2 AIs). Even pot plants labeled as “organically grown” (*n* = 13) were contaminated with 2.3 ± 2.4 AIs. The AIs azadirachtin, boscalid, difenoconazole, flonicamid, fluopyram, pyrethrine, spinosad, and spiroxamine were found on three or more pot plants labeled as “organically grown.” Only 6% of all pot plants and 0.4% of cut flowers were not contaminated with a pesticide (Table 1).

Most of the AIs detected in pot plants and cut flowers, namely, 59–60%, were not persistent, as they had a DT_50_ < 30 days according to the PPDB (Supplementary Table S4, Supplementary Fig. S2). Persistent or very persistent AIs, which remain in the field/soil for 100 or more days, were detected in 65% of all pot plants (*n* = 1000) and in 85% of all cut flowers (*n* = 237).

**Fig. 2:**
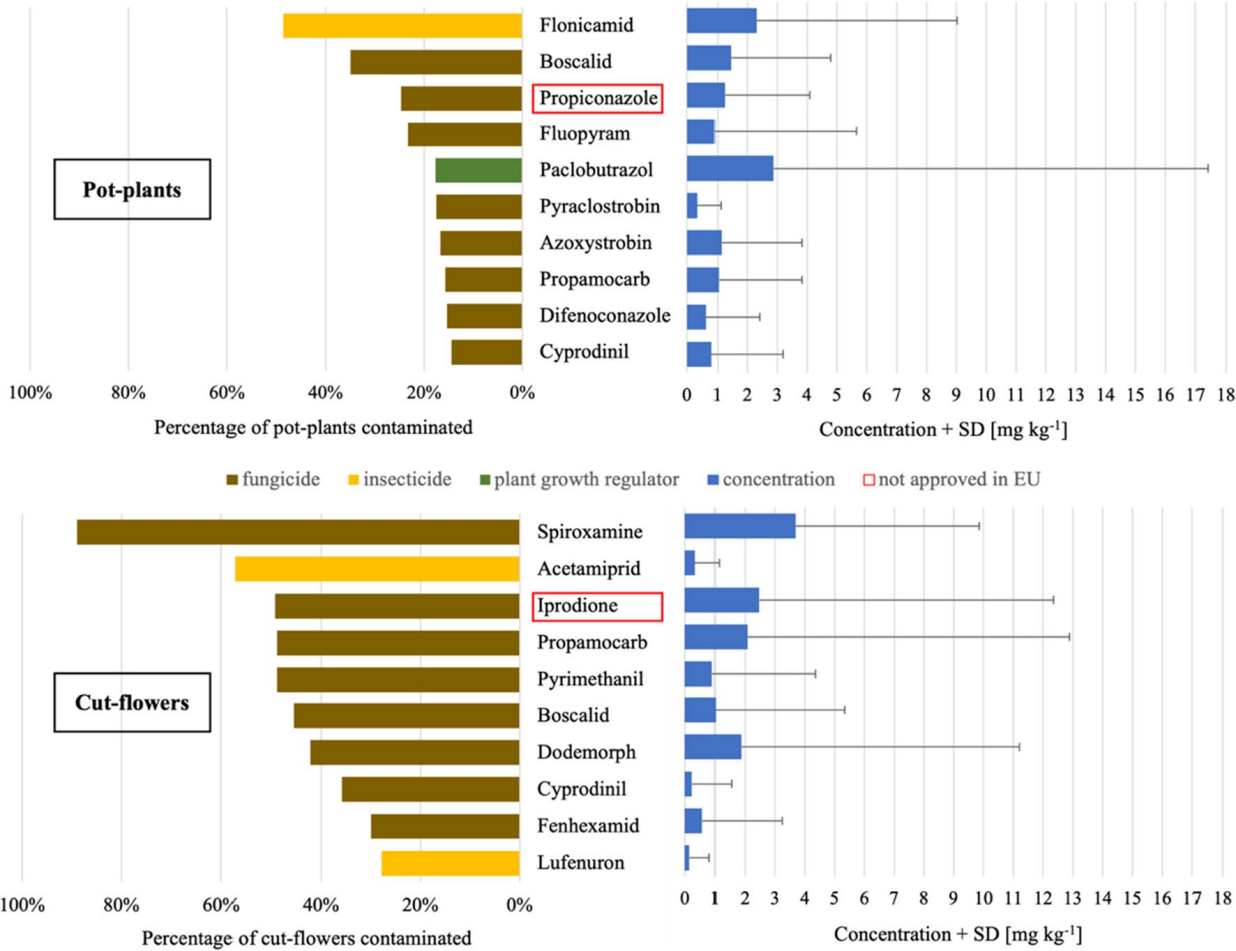
Top 10 most frequently detected insecticides, fungicides, and herbicides in pot plants (*n* = 1000) and cut flowers (*n* = 237) between 2011 and 2021and their concentrations. Pesticide names framed red had no EU approval in 2022. Color codes: brown…fungicides, yellow…insecticides, green…plant growth regulators

The substances most frequently detected in pot plants included the insecticide flonicamid and the fungicides boscalid, propiconazole, and fluopyram. Propiconazole has no longer been approved in the EU since December 2018 (EC 2024) and is classified as a highly hazardous pesticide (PAN 2021). On average, the 10 most common AIs in pot plants had half-lives between 3.1 and 254 days. Herbicides were only found up to 15 times in pot plants, but not in cut flowers. In cut flowers, fungicides and insecticides were most frequently detected with spiroxamine, acetamiprid, iprodione, and propamocarb being the most frequently detected AIs (Fig. 2). Among the 10 most common AIs detected in cut flowers, iprodione, fenhexamid, and lufenuron were considered highly hazardous (PAN 2021). Iprodione has no longer been approved in the EU since December 2017. The half-life of the 10 most frequent AIs in cut flowers ranged from 0.43 to 256 days. A list of the most common pesticides found in pot plants can be found in Supplementary Table S7 and of cut flowers in Supplementary Table S8.

### Toxicity to non-target organisms

The evaluation of the general ecotoxicity of AIs showed that regardless of acute or chronic exposure, at least 47% of the AIs detected in pot plants were highly or moderately toxic to honeybees, earthworms, birds, and mammals (Supplementary Fig. S3). Between 1 and 5% of AIs detected in pot plants had low acute and chronic toxicity to honeybees, earthworms, birds, and mammals. Overall, about 40% of AIs detected in pot plants showed a high contact and oral toxicity to honeybees and to birds. Contact exposure with abamectin, cyfluthrin, and deltamethrin and oral exposure with abamectin, fipronil, and imidacloprid showed the highest acute toxicity for honeybees; sulfoxaflor, carbaryl, and carbendazim for earthworms; and aldicarb, fenamiphos, and oxamyl for birds and mammals. In cut flowers, only 3% of the detected AIs were highly acutely toxic to earthworms. Toxic load (TL) calculations for honeybees, birds, and mammals were performed only for outdoor plants, while both indoor and outdoor plants were considered for calculating earthworm TLs. Of pot plants, a total of 872 plants were categorized for outdoor use and 128 plants for indoor use. All 237 cut flower samples were classified for indoor use only.

**Fig. 3:**
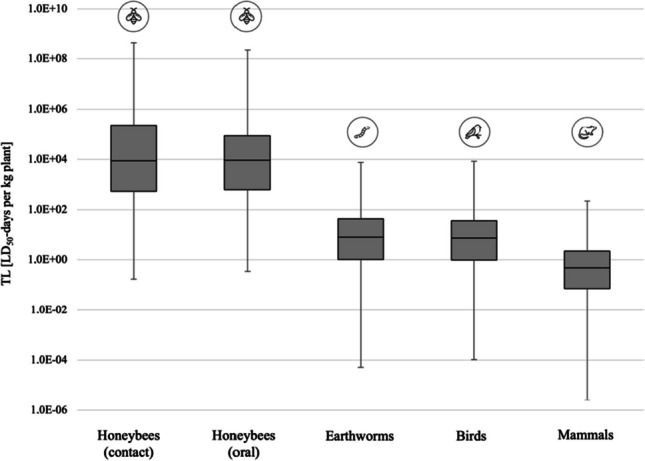
Toxic load (TL) based on LD_50_ values of AIs found in 1000 pot plants for different non-target organisms. Medians, minima, maxima, first, and third quartiles of TLs were calculated by excluding samples without AIs detected. Only pot plants for outdoor use were considered for TLs of honeybees (*n*_contact_ = 814; *n*_oral_ = 811), birds (*n* = 811), and mammals (*n* = 811). For earthworms, pot plants for both indoor and outdoor use were considered (*n* = 931). Note: *y*-axis is in logarithmic scale

The average TL per pot plant amounted to 1.6 * 10^6^ ± 16.5 * 10^6^ LD_50_-days kg^−1^ for honeybee contact exposure and 1.9 * 10^6^ ± 13.9 * 10^6^ LD_50_-days kg^−1^ for honeybee oral exposure; 64.3 ± 287.5 LD_50_-days kg^−1^ for earthworms; 62.9 ± 333.6 LD_50_-days kg^−1^ for birds; and 3.6 ± 14.4 LD_50_-days kg^−1^ for mammals (rats). The TLs for honeybees were much higher compared to those for other organisms, indicating that the AIs detected were much more toxic to honeybees than to other organisms (Fig. 3; Supplementary Fig. S4, Supplementary Fig. S5). The TLs for honeybees-contact and oral-toxicity were highly variable and ranged between 0.17 and 423 * 10^6^ LD_50_-days kg^−1^ for contact toxicity and 0.35 to 224 * 10^6^ LD_50_-days kg^−1^ for oral toxicity. For earthworms, TLs varied between 5.2 * 10^−5^ and 7525 LD_50_-days kg^−1^; for birds, between 1.1 * 10^−4^ and 8281 LD_50_-days kg^−1^; and for mammals, between 2.6 * 10^−6^ and 219 LD_50_-days kg^−1^. The toxic loads of plant species labeled as pollinator-friendly is shown in Supplementary Fig. S6.

For cut flowers, only the TL for earthworms was calculated (Supplementary Fig. S4, Supplementary Fig. S5). The TL per cut flower varied from 0.016 to 3210 LD_50_-days kg^−1^ with a median of 62.4 LD_50_-days kg^−1^. The average TL per cut flower is 184.8 ± 374.3 LD_50_-days kg^−1^. Comparing these values with the TL of the pot plants, it can be seen that the pot plant samples contained AIs with more variation in toxicity and a higher toxicity to earthworms than the AIs found in the cut flowers.

**Fig. 4:**
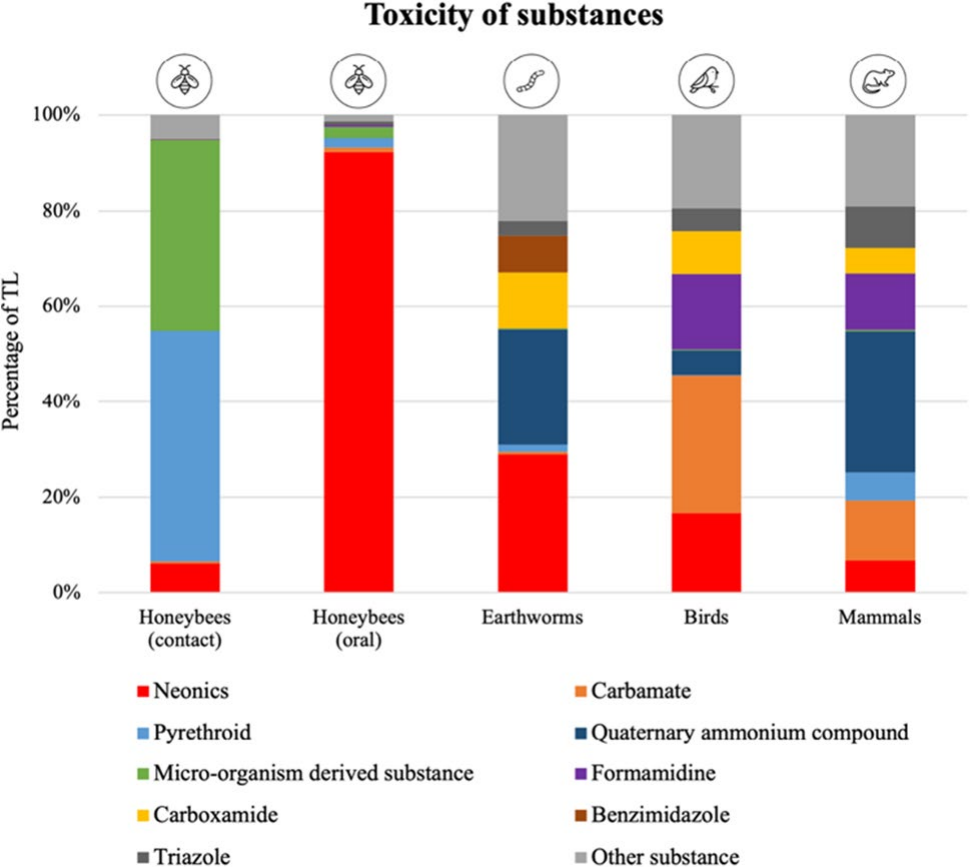
Contribution of the pesticide chemical classes to the toxic load (TL) for honeybees (contact and oral toxicity), earthworms, birds, and mammals calculated from active ingredients (AIs) detected in 1000 pot plants. For each organism, the top 5 substance classes contributing most to the TL were considered. To be included in this figure, the substance class had to contribute > 5% to the TL of at least one organism group; therefore, pyridazinone, pyrethroid esther, and strobilurin were not included

Neonicotinoids, pyrethroids, microorganism-derived substances (e.g., abamectin), compounds of quaternary ammonium, carbamate, formamidine, carboxamide, benzimidazole, and triazole contributed most to the TL calculations of the non-target organisms (Fig. 4). Neonicotinoids contributed to the TLs for all organisms, but mainly to honeybee oral toxicity (92% of TL_HB oral_). In contrast, pyrethroids and microorganism-derived substances contributed to the honeybee contact toxicity to a much higher extent (88%) than neonicotinoids (6%). Neonicotinoids (29%), quaternary ammonium and their compounds (24%), carboxamides (12%), and benzimidazole (8%) accounted for the highest proportion of TL for earthworms. In addition, AIs grouped together as “other substances” are responsible for about 22% of TL to earthworms. Compounds of quaternary ammonium, carbamate, and formamidine contribute the most (54%) to the TL for mammals. The TL for birds is mainly influenced by carbamates (29%), neonicotinoids (17%), and formamidine (16%).

### Toxicity to human health

**Fig. 5:**
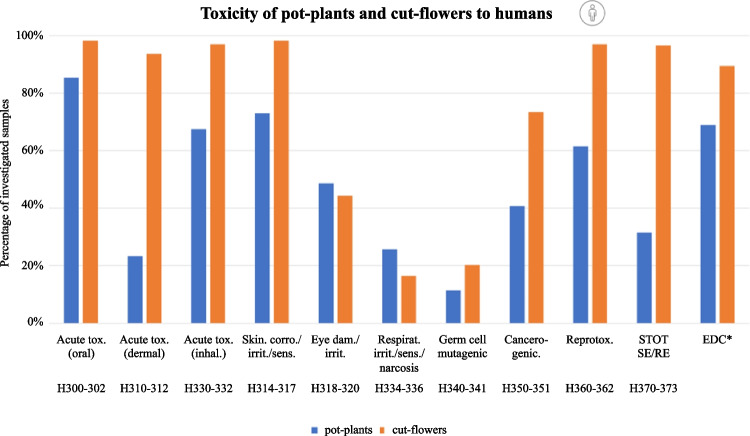
Proportion of human toxicological properties of AIs detected in 1000 pot plant samples and 237 cut flower samples. Classifications according to GHS hazard statements. *Endocrine disrupting chemicals (EDC) were assessed on the basis of the PPDB

Overall, only 8% of pot plants (81 spp.) and 1% of cut flowers (3 spp.) were without AIs with potentially harmful effects on human health. The pot plant species with most human health toxicity statements were golden cane palm (*Dypsis lutescens*; 11 ± 2.9 H-statements), lemon (*Citrus limon*; 8.1 ± 3.2 H-statements), and Christmas rose (*Helleborus niger*; 7.4 ± 4.5 H-statements). In total, 854 samples (85%) of pot plants contained at least one AI that was considered acutely orally toxic to humans (Fig. 5). Of the pot plants analyzed, 69% (689 samples) contained at least one AI with endocrine disrupting properties and 62% (615 samples) AIs with reproductive toxicity properties.

Of the cut flowers analyzed, 94–98% of the samples were contaminated with at least one AI with acutely oral, dermal, or inhalation toxicity; AIs that cause skin corrosion, irritation, or sensitization; and AIs with reproductive toxicity, or with specific target organ toxicity. About 89% (212 samples) and 73% (174 samples) of cut flowers contained AIs with at least one endocrine disrupting and cancerogenic AI. Respiratory irritation, sensitization, narcosis, and germ cell mutagenicity were the least common hazards (< 30% of samples) of AIs detected in pot plants and cut flowers.

AIs with dermal or inhalational toxicity or AIs causing skin corrosion/irritation/sensitization were found in 94–98% of cut flowers, and in 23%, 68%, and 73% of pot plants, respectively (Supplementary Fig. S7). The contribution of the detected fungicides, insecticides, and herbicides to human toxicological properties is shown in Supplementary Fig. S8.

The fungicide spiroxamine was one of the pesticides with the most human health toxicity statements. Despite approved in the EU until 2026, it is suspected of affecting the fertility of the unborn child (H361), is harmful to the skin (H312), harmful if swallowed (H302) and inhaled (H332), causes skin irritation (H315) and may cause an allergic skin reaction (H317). Spiroxamine was also among the most frequently detected AIs on cut flowers in this study (Fig. 2). Additionally, three AIs, namely iprodione, propamocarb, and propiconazole, with demonstrated endocrine-disrupting properties were among the 10 most common AIs in pot plants and cut flowers.

Poinsettia (*Euphorbia pulcherrima*) was the most contaminated plant species with 5.2 ± 3.3 AIs with an average concentration of 0.44 ± 11.7 mg kg^−1^ among 77 samples. The top-10 AIs in poinsettia were chlormequat, flonicamid, boscalid, fluopyram, propamocarb, pymetrozine, abamectin, propiconazole, pyraclostrobin, and spinosad (Supplementary Table S9).

### Countries of origin of ornamental plants

About 54% (544 samples) of all pot plants analyzed were grown in Europe, including Austria, Belgium, Denmark, France, Germany, Italy, the Netherlands, Poland, Portugal, and Spain. One sample was from Vietnam, the remaining 46% of pot plants (455 samples) were of unknown origin. Excluding the countries from which only one sample originated, pot plants from Germany had the highest average number of different AIs per plant (6.9 ± 4.2 AIs plant^−1^), while pot plants from Italy had the highest average pesticide concentration per plant (28.7 ± 123.0 mg kg^−1^; Supplementary Table S10).

Cut flowers came mainly (92%) from Africa, either Kenya or Tanzania. A small proportion was grown in North and South American countries (2%), more specifically Costa Rica and Ecuador, and in European countries (2%) such as Germany, Austria, and the Netherlands. Cut flowers grown in Ecuador were on average contaminated with the highest number of different AIs (20.0 ± 4.2 AIs plant^−1^). Flowers from Costa Rica had fewer different AIs (6.5 ± 4.9 AIs plant^−1^), but on average had higher pesticide contamination per plant (48.4 ± 23.1 mg kg^−1^; Table S8). However, it should be noted that for both countries, Ecuador and Costa Rica, only two plants were analyzed.

AIs not authorized in the EU were found in 60% (603 samples) of all pot plants and in 85% (201 samples) of all cut flowers. More than half of all pot plants (54%) in which unauthorized AIs were detected were cultivated in Europe. The origin of the other half (45%) is unknown. The residues found included AIs like aldicarb, bifenthrin, carbaryl, and carbendazim, which are classified as highly hazardous pesticides according to the Pesticide Action Network (PAN 2021). The majority (93%) of cut flowers with residues of AIs not approved in the EU came from African countries; a few came from North and South America (2%) and Europe (1%). Around 4% of the cut flowers with AIs not approved in the EU had an unknown country of origin.

## Discussion

Our study shows that ornamental plants can act as vectors for pesticide contamination in homes and gardens. Placing pesticide-contaminated pot plants in the garden or composting wilted cut flowers can pose a hazard to non-target organisms that feed on these plants or their remains. It was worrying to see that even plants labeled as bee-friendly contained pesticide residues that were harmful to bees. A high proportion of the ornamental plants examined also contained pesticides associated with hazards for human health. At this point, it is important to emphasize that our approach to assessing ecotoxicological effects is simple, as the actual exposure of non-target organisms to pesticide residues in pot plants and cut flowers is much more complex. Our results also suggest that the discussion about the ecotoxicological effects of pesticides and their residues should increasingly include the production and distribution of ornamental plants (Zaller 2020).

We found that 94% of ornamental plants contained pesticide residues, which is similar to the proportions found in other studies (Global 2000 2021, 2022, Greenpeace 2014; Lentola et al. 2017); see also Supplementary Table S11. Furthermore, we found that 84% of plant samples contained more than one AI, which was also in the range of previous studies (Global 2000 2021, Greenpeace 2014; Lentola et al. 2017). The presence of such pesticide cocktails of multiple AIs on plants is of concern as they are still not well understood and neglected in current risk assessments (Belden & Brain 2018; Geissen et al. 2021; Goulson et al. 2015). Studies analyzing these cocktail effects report a great increase in ecotoxicity with simultaneous presence of more than one pesticide (Belden & Brain 2018; Giorio et al. 2017; Lentola et al. 2017), impacts on honeybee populations (Taenzler et al. 2023; Zhu et al. 2014), earthworms and soil functions (Froger et al. 2023; Teng et al. 2022; Zaller et al. 2021), birds (Moreau et al. 2022), or rats (Mesnage et al. 2023; Rizzati et al. 2016).

Two studies with a comparable research design were found for cut flowers. In these studies, 50 and 90 bouquets of cut flowers sold in Belgian cities were analyzed, and 97 and 107 different AIs were detected (Toumi et al. 2016a, b). With 99–100% of plants containing at least one AI and an average of 10–14 AIs per bouquet, the contamination was similar to our study. The AIs most frequently found in cut flowers in previous studies (Toumi et al. 2017, 2016b) and the current study were the fungicides spiroxamine, boscalid, and iprodione. Generally, fungicides are most commonly used in cut flower production, as fungal diseases such as *Botrytis cinerea*, which cause gray mold, are a constant threat to cut flowers (Muñoz et al. 2019; Vega et al. 2020). With increasing fungicide treatments, fungi have started to develop resistance to AIs, and four of the most frequent AIs detected in cut flowers in the current study (boscalid, iprodione, cyprodinil, and fluopyram) were also among the AIs for which fungal resistance was identified in cut-roses (Muñoz et al. 2019).

The finding that 60% of pot plants and 85% of cut flowers contained AIs not approved in the EU can have several reasons: (i) it could be imports of contaminated plants from countries with lower standards than in the EU (Sarkar et al. 2021); (ii) the domestic application of unapproved pesticides that were illegally imported, stockpiled, or temporarily authorized through so-called emergency permits (Zaller 2020); (iii) the pesticides in question have long half-life and were applied when use was still permitted; (iv) the pesticides were still authorized during the sampling period (before 2022), or there was still a grace period for the pesticides; or (iv) the contamination originates from sources other than the floriculture sector (Linhart et al. 2019, 2021; Zaller et al. 2022). In addition, some AIs may never have been authorized in the EU because their market potential in Europe is limited or the pests, fungal diseases or weeds they control are not relevant in Europe.

### Effects on honeybees

We found that 4 out of 10 pot plants contained AIs that are considered harmful to honeybees. This is concerning as honeybees are surrogate species to evaluate pesticide effects for insect pollinators in environmental risk assessments and because populations of insect pollinators have dramatically declined in recent decades (Donkersley et al. 2020; Goulson et al. 2015; Nicholson et al. 2024). As awareness of this problem has grown, garden stores have begun selling ornamental plants labeled “pollinator-friendly” (RHS 2023; Seitz et al. 2020), which are intended to provide nectar or pollen, improve pollinator health, and prevent declines in pollinator abundance and diversity (Kalaman et al. 2020). According to the Royal Horticultural Society (RHS 2023) 306 of the 1000 pot plants sampled (31%) could be classified as “pollinator-friendly.” However, these pollinator-friendly plants contained on average 5.9 ± 4.1 AIs per plant, about 13% of them contained more than 10 AIs; Christmas rose (*Helleborus niger*) contained the maximum of 21 AIs per plant. Only 8% (23 samples) of the pollinator-friendly pot plants contained no pesticide residues. About 19% (58 samples) of the pollinator-friendly plants analyzed were contaminated with pyrethroids, 18% (55 samples) with neonicotinoids, and 2% (7 samples) with chlorpyrifos. The systemically acting neonicotinoids are particularly problematic, as they have many harmful effects on honeybees and wild bees (Fischer et al. 2014; Rundlöf et al. 2015) and the use of three neonicotinoid AIs (imidacloprid, thiamethoxam, clothianidin) on open-field crops was therefore completely banned in the EU in 2018 (Sgolastra et al. 2020). While the potential hazards of neonicotinoids for honeybees can be severe, wild bee species are expected to be even more at risk from this exposure (Nicholson et al. 2024). Very little information is available from studies on ornamental plants, but it has recently been shown that the application of imidacloprid to ornamental pot plants strongly reduces foraging activity and reproduction of the solitary bee species *Megachile rotundata* (Cecala & Wilson Rankin 2021). A study has also shown that pesticide residues can spread between flowers, leaves, roots, and soils of ornamental plants and thus affect non-target organisms above and below ground (Porseryd et al. 2024).

Insecticides with an LD_50_ less than 1 μg bee^−1^ pose a major hazard to honeybees and are therefore classified as “highly toxic” to bees by the PPDB (Lewis et al. 2016). We found 25 such highly toxic insecticides in 118 (39%) different pollinator-friendly pot plants, including AIs such as abamectin, clothianidin, imidacloprid, indoxacarb, spinosad, and thiamethoxam. Among the 10 most abundant pollinator-friendly pot plants in our dataset, *Chrysanthemum indicum* contained AIs with the highest average toxic load for honeybee contact toxicity (4.3 * 10^6^ ± 13.5 * 10^6^ LD_50_ dosages), whereas *Helleborus niger* contained AIs with the highest average toxic load for honeybee oral toxicity (6.6 * 10^6^ ± 14.2 * 10^6^ LD_50_ dosages). The majority (55%) of all pollinator-friendly pot plants were grown in European countries such as Austria, France, Germany, Italy, and the Netherlands. For the remaining plants, the country of origin was unknown.

The current results are in line with those from previous studies, according to which plants labeled as pollinator-friendly are contaminated with multiple AIs and some even contained pesticides that are highly toxic to pollinators (FoE 2014, Global 2000 2022, Greenpeace 2014, Lentola et al. 2017). While such a label only indicates whether a plant species is attractive to pollinators, consumers may assume that the label implies that the specific plant they are buying is actually safe for pollinators and without pesticide contamination. The choice of ornamental plants can have a major impact on the abundance and diversity of flower-visiting insects in a garden (Palmersheim et al. 2022), and the introduction of pesticide-contaminated plants could be a trap for them.

### Effects on earthworms

Earthworms could come in contact with pesticides contained in pot plants and cut flowers when plants are planted in flower beds or composted after use. AIs in pot plants had a theoretical TL for earthworms equal to 64.3 LD_50_ days per kg plant material, which is equivalent to the TL found for birds, but more than 30,000 times smaller than the TL for honeybees. Insecticides and fungicides were the most frequently detected AIs in this study, and these pesticide classes have also been shown to be among the most toxic to earthworms with effects on behavior, reproduction, and mortality (Pelosi et al. 2014). While neonicotinoids have been mainly discussed in terms of their effects on non-target insects, they have also been shown to have direct and/or indirect impacts on the growth, burrowing activity, and reproduction of earthworms (Capowiez et al. 2006, 2005; Faheem & Khan 2010; Fernández-Gómez et al. 2011; Wang et al. 2019). Neonicotinoids can also cause changes in various soil functions (van Hoesel et al. 2017; Zaller et al. 2016) and can even lead to outbreaks of secondary pests, e.g., populations of two-spotted spider mite (*Tetranychus urticae*) (James & Price 2002); however, this has not been studied in the context of ornamental plants and consequences for ecological interactions in the garden ecosystem.

The toxicity of pesticide residues in ornamentals to earthworms depends on the persistence of AIs and their concentrations at disposal. Cut flowers have a postharvest vase life between 5 days and 4 weeks (Nguyen & Lim 2021). Assuming that pesticide application takes place until after harvest (Toumi et al. 2017), transportation of the flowers to their destination within 1 day (Podofillini et al. 2015) and 5 days before cut flowers are disposed of or composted, the pesticides must have a half-life of at least 6 days to still be present with 50% concentration on cut flowers at the time of composting. In the current dataset, around 76% of the AIs detected had a half-life > 6 days, with an average half-life of 65 days in cut flowers. Of the highly acutely toxic AIs to earthworms, only two had a half-life of more than 6 days (carbaryl with a DT_50_ of 16 days, and carbendazim with a DT_50_ of 22 days). The timing of exposure also plays a role, as decomposition takes 45–60 days (Etheredge & Waliczek 2022), leading to a potential chronic exposure of earthworms to pesticides for up to 8.5 weeks.

Assessing the toxicity of pesticides in pot plants is a challenge due to the large differences in life span after harvest. Therefore, very few studies have investigated the effects of pesticide residues on earthworms and the quality of compost soil from ornamental plants. Composting can reduce the toxicity of pesticides through mineralization, compound breakdown, volatilization, and bio-transformation (Etheredge & Waliczek 2022). When the quality of vermicompost from cut flower waste was analyzed, residues of clopyralid, clofentezine, and lufenuron were detected in the resulting compost, although the residue levels were in line with industry standards (Etheredge & Waliczek 2022). Numerous adverse effects of pesticide residues to earthworms have been identified (Jeyaprakasam et al. 2021), although earthworms have also been shown to have some tolerance to pesticides (Pelosi et al. 2014; Sharma et al. 2019). In addition to earthworms, recent reviews and meta-analyses have shown that pesticides reduce the abundance and diversity of soil fauna communities (Beaumelle et al. 2023; Gunstone et al. 2021). However, it should also be considered that earthworms living in a compost heap most likely do not feed exclusively on pesticide-contaminated ornamental plants, but also on uncontaminated organic material. It has been shown that the number of pesticides in the individual plant parts of ornamental pot plants varies, with more pesticides in the soil than in the roots, leaves, or seeds (Porseryd et al. 2024), but the effects on earthworms or other soil organisms in ornamental plants have not yet been investigated.

### Effects on birds

To our knowledge, this is the first study to investigate the potential exposure of birds to pesticides detected in ornamental pot plants. Our results show that pesticides detected in pot plants contained a toxic load equivalent to 63 LD_50_ days kg^−1^ for house sparrows, which is higher than the TL calculated for mammals, similar to the TL for earthworms but well below the TL for honeybees. Of the detected AIs, carbamates, neonicotinoids, formamidine, and carboxamide are considered very toxic to house sparrows. This is partly consistent with other studies showing that, in addition to organophosphates and conazoles, neonicotinoids and carbamates have been associated with death or sublethal effects in birds (Katagi & Fujisawa 2021; Rogers et al. 2019; Tassin de Montaigu & Goulson 2020). The consumption of four seeds contaminated with neonicotinoid imidacloprid over 3 days was sufficient to influence the migration behavior of white-crowned sparrows, *Zonotrichia leucophrys* (Eng et al. 2017), and the ingestion of field-realistic levels of imidacloprid reduced feeding and delayed departure from stopover sites with population-level consequences (Eng et al. 2019).

In general, songbirds such as sparrows are more sensitive to pesticides as they are comparatively smaller than bird species used in official pesticide risk assessments (Katagi & Fujisawa 2021). Among pesticides used in UK agriculture the plant growth regulator chlormequat was the most toxic to corn buntings, *Emberiza calandra* (Tassin de Montaigu & Goulson 2020), among herbicides used in Austrian agriculture diquat was the most toxic to European serin (*Serinus serinus*) (Cech et al. 2022).

It remains an open question to what extent pesticide residues in pot plants pose a real threat to birds in residential gardens. Inhalation exposure occurs mainly during pesticide spraying, suggesting a threat mainly during the cultivation of ornamental plants or when home gardeners apply AIs themselves (Sánchez-Bayo et al. 2002). Exposure of birds via digestion has been studied mainly in agricultural settings, where birds may eat pesticide-treated seeds. However, this could also be the case when systemic pesticides such as neonicotinoids have been detected in ornamental plants (Eng et al. 2017; Fernández-Vizcaíno et al. 2022; Tassin de Montaigu & Goulson 2020; Vyas et al. 2007). Dermal contact is another route of pesticide exposure for birds in residential gardens; however, this has not been investigated so far. Since ornamental plants are treated until after harvest (Toumi et al. 2017), pesticide exposure through contact in residential gardens is possible, depending on the persistence of applied AIs. Another possibility of exposure is the trophic transfer of pesticides, e.g., when birds feed on herbivorous insects on contaminated ornamental plants (Baudrot et al. 2020). While toxic loads in the current study were calculated based on acute oral LD_50_ values available in the PPDB, future toxic load calculations should also consider contact LD_50_ values to account for dermal exposures as well.

### Effects on mammals other than humans

The pesticide residues detected in pot plants were the least toxic to mammals compared to the other species investigated: TL amounted to 3.6 LD_50_ days to rats per kg pot plant (no TL calculation was made for cut flowers). The substances contributing most to TL in the current study were quaternary ammonium compounds, carbamates, formamidines, triazoles, neonicotinoids, and pyrethroids (75% of this TL). Chlormequat is a commonly applied plant growth regulator used in ornamental plant production to improve flowering and reduce shoot growth (Parlakova Karagöz & Dursun 2022; Sevim et al. 2021). While chlormequat is moderately toxic to mammals, negative effects on reproduction in pigs have been reported (Sørensen & Danielsen 2006). Neonicotinoids have lower toxicity to mammals compared to insects (DiBartolomeis et al. 2019); however, they are still considered moderately to highly hazardous to mammals (Rogers et al. 2019). Exposure of white-tailed deer to imidacloprid has shown to reduce jawbone length, body mass, activity levels, organ size, and fawn survival (Royte 2021). The disadvantage of using systemic insecticides such as neonicotinoids in ornamental plant systems are therefore unintended effects on beneficial and non-target organisms (Cloyd et al. 2011). In addition, the highly water-soluble neonicotinoids can be leached of pot plants during irrigation or rainfall, but this has not yet been scientifically investigated.

Mammals living in residential gardens, such as hedgehogs, rabbits, mice, moles, foxes, domestic cats, and dogs, are potentially exposed to pesticides in ornamental plants. However, similar to bird exposure, the routes and extent of mammalian exposure to pesticide residues in ornamentals are not well studied. Possible exposure may also occur via trophic transfer of pesticides through predator–prey interactions (Baudrot et al. 2020), e.g., when a hedgehog eats an earthworm that has been feeding on composted cut flowers. Such trophic transfers have been studied especially with anticoagulant rodenticides (Coeurdassier et al. 2014), where consumption of contaminated voles reduced the abundance of foxes (Jacquot et al. 2013) or affected mustelid populations (McDonald et al. 1998). Broad-spectrum pesticides used in agriculture and forestry also affect the profile of pesticide residues in wild boar, roe deer, and deer (Kaczyński et al. 2021). Overall, more attention needs to be paid to the ecotoxicological impacts of pesticide use in ornamental horticulture (Yin et al. 2023) and to potential impacts of pesticide contamination by ornamental plants in gardens, as these are often important habitats (Vitorino et al. 2021).

### Potential hazards for human health

Dermal and airborne exposure is considered to be the main route by which humans come into contact with pesticides in the ornamental plant life cycle, as farmers and residents in the growing area (Friedman et al. 2020; Lu 2007), florists, or gardeners (Bouvier et al. 2006; Toumi et al. 2016a, b). Among the human health effects associated with flower production areas, the following should be emphasized: neurological toxicity (Abdel Rasoul et al. 2008; Grandjean et al. 2006; Handal et al. 2007b; Harari et al. 2010), decreased fertility (Handal et al. 2008; Lauria et al. 2006), and respiratory problems (Hanssen et al. 2015).

The pesticides detected in ornamental plants in the current study were associated with hazards to human health: 47% of pot plants and 48% of cut flowers contained AIs with acute oral toxicity, and 28% of pot plants and 34% of cut flowers contained AIs that were acutely toxic by inhalation. Between 33 and 37% of the AIs detected in pot plants and cut flowers, respectively, were categorized as endocrine disrupting chemicals. Less than 20% of the AIs found in pot plants and cut flowers were acutely toxic regarding dermal contact, eye-damaging or irritating, respiratory irritating, mutagenic to germ cells, cancerogenic, toxic to reproduction, or exhibited specific target organ toxicity upon single or repeated (STOT SE/RE). In another study, acute dermal toxicity of more than 40% of AIs detected in cut flower samples without analyzing pot plants (Toumi et al. 2016b). Overall, the AIs found in pot plants showed a similar toxicity to humans as those found in cut flowers, with the largest difference of AIs in cut flowers being higher in acute inhalation toxicity. Pot plants and cut flowers are therefore treated with similarly toxic AIs. The pesticides with the highest number of H-statements and therefore the highest potential toxicity to human health were the insecticides emamectin benzoate and methoxychlor, the fungicide spiroxamine for pot plants, the fungicides fenpropidin and spiroxamine, and the insecticide emamectin benzoate for cut flowers.

When analyzing the effects on human health of the top-3 pesticide types detected in pot plants—insecticides, herbicides, and fungicides—it was seen that insecticides most frequently exhibited acute oral toxicity and endocrine-disrupting properties. In addition, 39% of insecticides were acutely toxic when inhaled and 23% when absorbed through the skin. The herbicides detected in pot plants were mainly acutely oral toxic (32%), endocrine disrupting (29%), and cancerogenic (25%). Fungicides, on the other hand, were mainly endocrine disrupting (37%), acutely oral toxic (34%), and causing skin corrosion, irritation, or sensation (31%). Fungicides were most frequently toxic to reproduction, while herbicides were mainly cancerogenic. Among pesticide categories detected, insecticides appear to pose the highest potential hazard to human health, which is consistent with the results of previous studies (Boedeker et al. 2020; Cech et al. 2023).

Endocrine disrupting chemicals interfere with (human) hormones required for homeostasis, reproduction, development, and behavior (Marlatt et al. 2022), and have been linked to neurological damage, diabetes (Ismanto et al. 2022), prematurity, reduced semen quality, breast and prostate cancer, and attention-deficit disorders (Kahn et al. 2020). These effects have been shown at very low concentrations (McKinlay et al. 2008) and can be hazardous to human health and wildlife (Marlatt et al. 2022). Previous studies found that 86% of pot plant and cut flower samples contained AIs that are cancerogenic, toxic to reproduction, endocrine disrupting, organ damaging, or classified as highly dangerous by the United Nations World Health Organization (Global 2000 2022).

Since we only determined the remaining pesticide residues at the time of sale, it can be assumed that pesticide exposure was much higher during cultivation of ornamental plants in the field. Indeed, workers in the floricultural industry showed a high pesticide exposure (Gelaye 2023; Pereira et al. 2021; Toumi et al. 2017) with 37 AIs detected on florists’ gloves and 5 AIs exceeding the acceptable operator exposure levels AOELs (Toumi et al. 2017). For floricultural workers, exposure to pesticides has been associated with genetic damage, premature births and malformation of infants, fever, dizziness, cancer (Toumi et al. 2017), effects on neurobehavioral development in children (Handal et al. 2007a), Alzheimer’s disease, increased cases of depression, and even suicide (Gelaye 2023). Although the use of appropriate protective equipment (e.g., gloves, face shields) can considerably reduce the risk of dermal exposure in workers and recreational gardeners (Toumi et al. 2017), there is often a lack of knowledge about the importance of wearing this equipment (Bouvier et al. 2006; Gelaye 2023; Toumi et al. 2016b).

While there are AOELs for occupational pesticide exposure of humans, there are no regulatory limits that protect private individuals from potentially hazardous pesticide residues in ornamental plants. Some authorities (e.g., the EU, U.S., Japan, and Hong Kong) have adopted regulations for the trade of flowers and foliage to ensure the esthetics of flowers and the absence of pests and pathogens upon import (Pereira et al. 2021). However, no country or region has set maximum residue limits (MRLs) for pesticides in ornamental plants other than edible plants and plants used for infusions such as chamomile or as animal feed (Pereira et al. 2021).

The impact of pesticide use in ornamental plant production on the environment is beyond the scope of the current study. However, studies have reported contamination of water resources around flower crops with impacts on aquatic organisms (Breilh 2012; Jansen & Harmsen 2011), soil health (Aguirre 2003), and atmospheric transport of pesticides into nature reserves (Dunn et al. 2014).

### Countries of origin of ornamentals

We found that pot plants from Germany and Italy and cut flowers from Ecuador and Costa Rica had the highest number and concentration of pesticide AIs. In contrast, pot plants from Austria and cut flowers from the Netherlands were the least contaminated in terms of the number and concentration of detected AIs. This is consistent with other studies showing that cut flower samples from Germany were the most contaminated and samples from the Netherlands with the lowest average AI levels (Toumi et al. 2016b). However, cut flowers from the Netherlands were also found with numbers of AIs not approved in the EU (Toumi et al. 2016a). Our and previous findings show the intensive use of pesticides in floriculture (Pereira et al. 2021), with examples of daily pesticide use on Ethiopian cut flower farms (Mengistie et al. 2017).

The results of the current study show that pot plants in German and Austrian garden centers originate mainly from Europe (Germany, Netherlands, Austria, Italy, and Denmark) or have an unknown country of origin. However, it is unclear whether the European pot plants sampled for this study originally came from African or Central American cuttings (Havardi-Burger et al. 2020). The cut flowers originated mainly from Kenya and Tanzania, and—in small numbers—from Central and South America (Costa Rica and Ecuador) and Europe (the Netherlands), partly reflecting global trend of increasing exports from Ecuador, Kenya, and Ethiopia (Pereira et al. 2021). The detection of unauthorized pesticides in ornamental plants in our study was independent of the origin of the pants. Originally, we had expected that imports from countries where standards are lower than in the EU would contain more AIs banned in the EU (Sarkar et al. 2021). Similarly, other studies found unapproved pesticides in cut flowers grown in Belgium, the Netherlands, and Italy (Pereira et al. 2021; Toumi et al. 2016b). One explanation for the unapproved AIs found could also be the long sampling period of our study. Since EU legislation from 2022 was used for this study, some AIs may have been approved in the EU at the time of sampling and analysis.

### Study limitations

Our study has answered the research questions, but their remain limitations and areas for further investigation. First, the results only provide information on the potential hazards to non-target organisms and humans, but not on the quantitative exposure, as we do not know how much of the pesticide residues would actually be taken up by the organisms. In addition, toxicity varies depending on developmental stage of the organism and feeding behavior. However, our main aim was to investigate pesticide residues contained in ornamental plants and how this might affect biodiversity. Secondly, whole plants were analyzed in this study, so we cannot say to what extent leaves, flowers, pollen, or nectar contain pesticide residues. This would be important to assess the actual threat to honeybees and other pollinating insects. Earthworms in a compost heap would also not feed on compost consisting exclusively of contaminated ornamental plants. Seed eating or insectivorous birds would only take up a tiny fraction of the pesticides contained in ornamental plants. Predatory mammals could come into contact with pesticide residues through trophic transfer if they consume herbivorous prey that has fed on ornamental plants. However, such trophic transfers of pesticides have rarely been investigated so far. Third, the dataset did not allow a longitudinal study of pesticide residues and toxicity over time, as the number of samples and plant species analyzed changed annually. Fourth, we did not consider the effects of multiple pesticide residues present in ornamental plants, which could influence the calculated TLs and ecotoxicological effect. We are aware that our analysis cannot capture the complexity of the potential ecotoxicological effects of pesticide residues in ornamental plants. Nevertheless, our findings show that pesticide residues in ornamental plants can pose an ecotoxicological hazard and that different organismic groups are affected differently.

## Conclusions

Our study showed that the majority of pot plants and cut flowers contained several pesticide residues that are transferred to private households and gardens. The ecotoxicological assessment of the detected pesticides based on LD_50_ values showed that AIs in pot plants were most problematic for honeybees and much less problematic for earthworms, birds, and mammals. Remarkably, a high percentage of ornamental plants also contained AIs with hazardous properties for humans, such as reproductive toxicity, carcinogenicity, or endocrine disruption. However, these results only indicate potential toxicities to biodiversity and humans, and the actual exposure and effects remain unknown. Nevertheless, it can be assumed that most of these hazardous pesticides were used in the cultivation of ornamental plants, which has far more harmful effects on biodiversity, farmers, and local residents in the cultivation area.

The high detection of pesticide AIs in ornamental plants poses a threat to non-target organisms, including humans working with these plants. However, to better understand the actual risks and impacts of contaminated ornamentals, further research is needed that links the use of pesticides in floriculture with the fate of contaminated ornamentals, their impact on above and belowground non-target organisms in gardens with health data of farmers, residents, and residential gardeners. Unlike in agriculture, there are still no globally harmonized maximum residue levels (MRLs) for ornamental plants that could help reduce the use of toxic chemicals in the production of ornamentals (Pereira et al. 2021). In addition, the strict regulation of pest control on imported flowers could also a reason for the excessive use of pesticides in floriculture (Pereira et al. 2021). Minimizing the use of pesticides in ornamental plant production would also reduce aquatic ecotoxicity (Klátyik et al. 2024; Yin et al. 2023), which was not considered in the present study.

While the use of pesticides in agriculture and its consequences have been widely researched, more knowledge is necessary about pesticide use in the production of ornamentals and their effects on the health of ecosystems and humans. Studies have shown that greenhouses used for production of ornamental flowers are associated with larger pesticide emissions than greenhouse production of vegetable crops, because of a higher frequency of pesticide applications and a higher number of AIs used (Boye et al. 2022).

In order to avoid the introduction of pesticides into private gardens, ornamental plants could be purchased that were grown without the use of synthetic pesticides. Surprisingly, however, we found that even some organic ornamental plants contained pesticide residues. The source of this contamination remains unclear as the detected AIs are not permitted in organic floriculture. In addition, ornamental plants labeled as “pollinator-friendly” were found to contain bee-toxic residues, which calls for better control of these labels. This is particularly important as the use of the term “bee-friendly” had the greatest economic value as it had the highest willingness-to-buy (Wollaeger et al. 2015). This argues for better monitoring of pesticides in floriculture, including production, transport, and storage, to ensure that the regulatory process sufficiently limits collateral damage to the environment and human health from the use of pesticides in ornamental plant production.

### Supplementary Information

Below is the link to the electronic supplementary material.

Supplementary file1 (DOCX 950 KB)

Supplementary file2 (XLSX 11 KB)

Supplementary file3 (XLSX 135 KB)

Supplementary file4 (DOCX 87 KB)

## Data Availability

Datasets used and/or analyzed during the current study are available from the corresponding author on reasonable request.
