# Supplementary material for: Ornamental plants as vectors of pesticide exposure and potential threat to biodiversity and human health
Source: Environ Sci Pollut Res Int. 2024 Jul 24;31(36):49079–99. doi: 10.1007/s11356-024-34363-x (PMC11310276; doi:10.1007/s11356-024-34363-x)
Supplement: Supplementary file 1 — Supplementary file1 (DOCX 950 KB) [file 11356_2024_34363_MOESM1_ESM.docx]

**Supplementary Figures**

Manuscript Chwoyka et al: Ornamental plants as vectors of pesticide exposure and potential threat to biodiversity and human health.


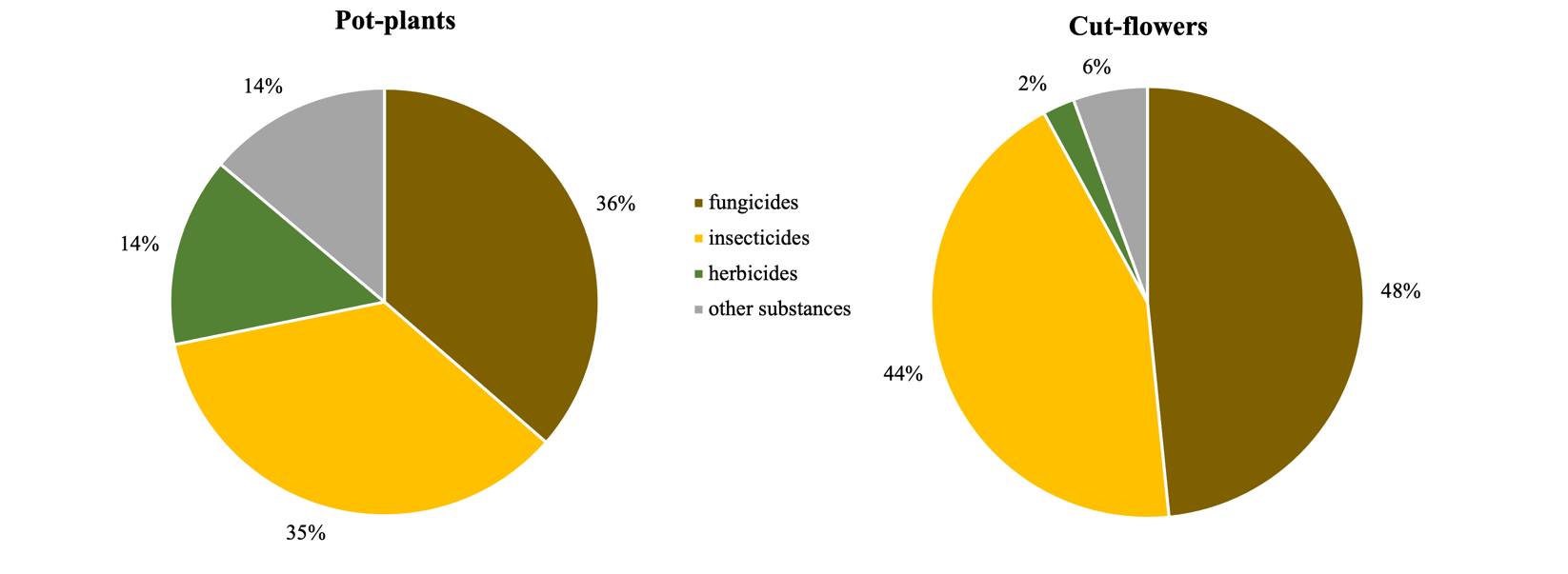


**Fig. S1.** Pesticide types detected on pot-plants (n=195) and cut-flowers (n= 126). Other substances found on pot-plants included metabolites, plant growth regulators, nematicides, repellents, acaricides, and biocides. Other substances found on cut-flowers included acaricides, biocides, and nematicides.

**Fig. S2.** Persistence of AIs on pot-plants (n=1,000) and cut-flowers (n=237), evaluated based on the precautionary principle (i.e., AI with highest persistence used for categorization of plant).


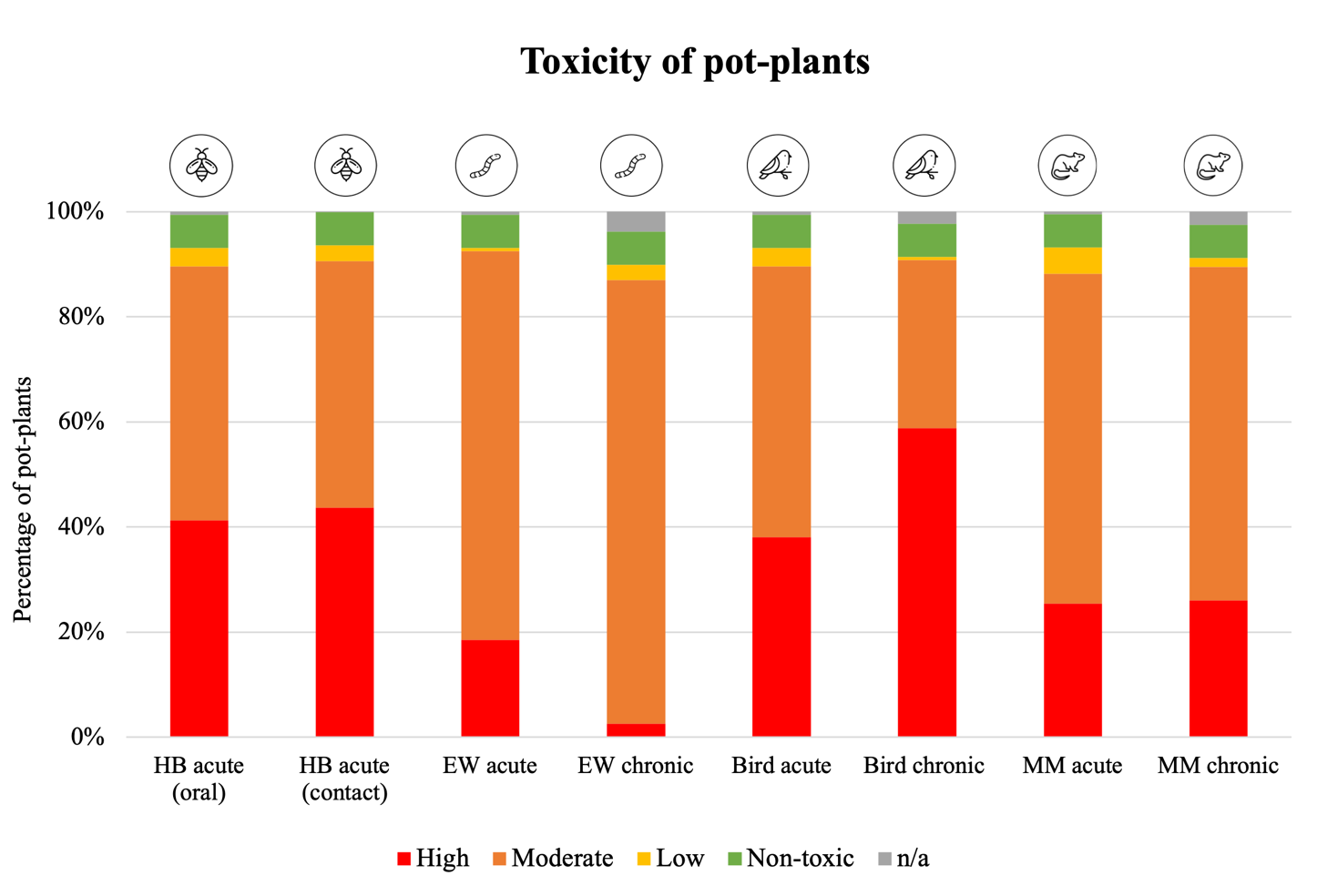


**Fig. S3.** Toxicity of pot-plants (n=1,000) for honeybees, earth worms, mammals, and birds based on LD_50_, LC_50_, NOEC, NOEL and NOAEL values. If several AIs were detected on a plant, the one with the highest toxicity was used for classifying the toxicity rating.


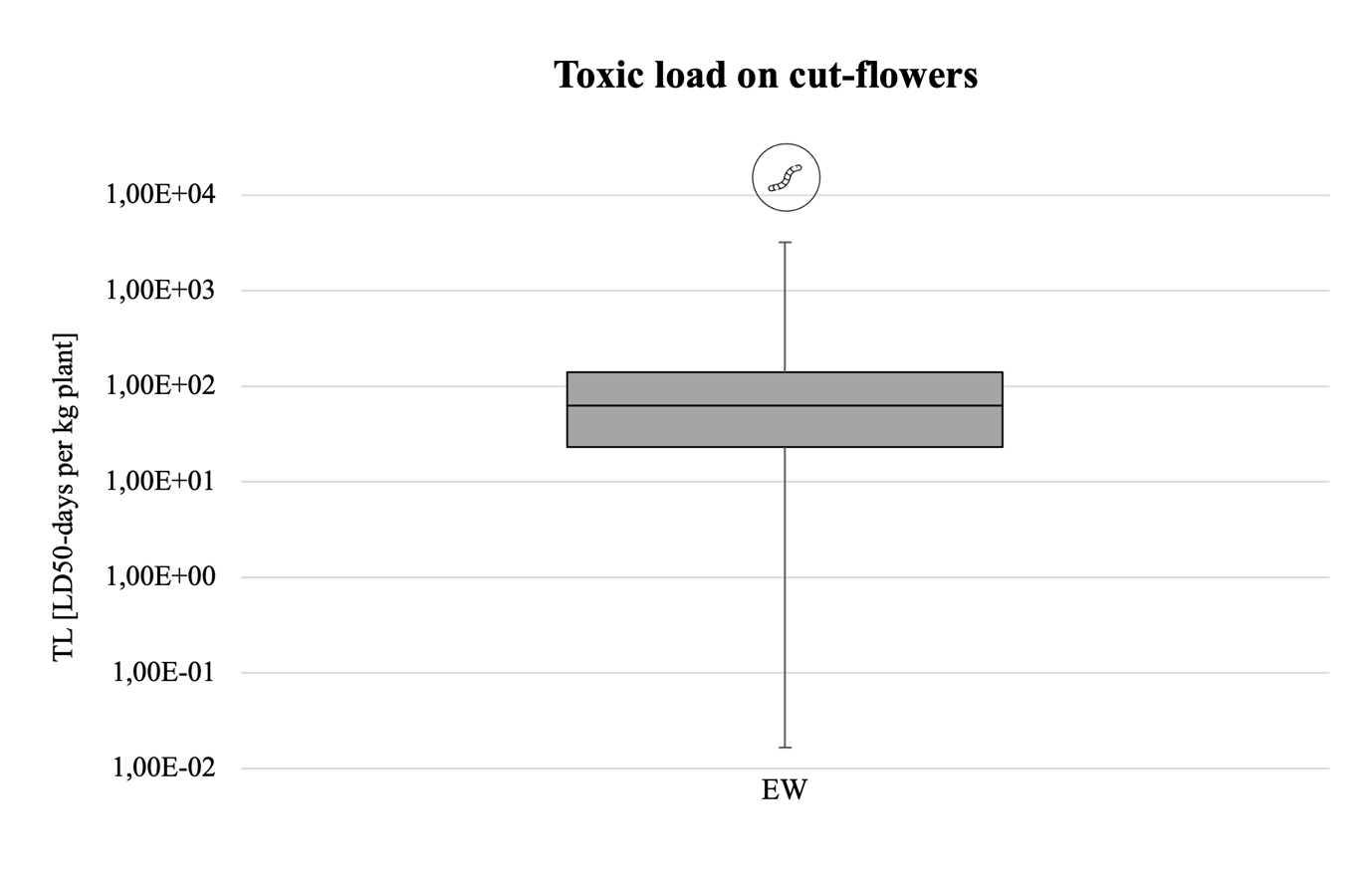


**Fig. S4.** Boxplot representing TLs found on cut-flowers for earthworms. Minima, maxima, first, and third quartile of TLs were calculated by excluding zeros (= uncontaminated plants) (n_cf_ _EW_ = 236). Note: y-axis is in logarithmic scale.


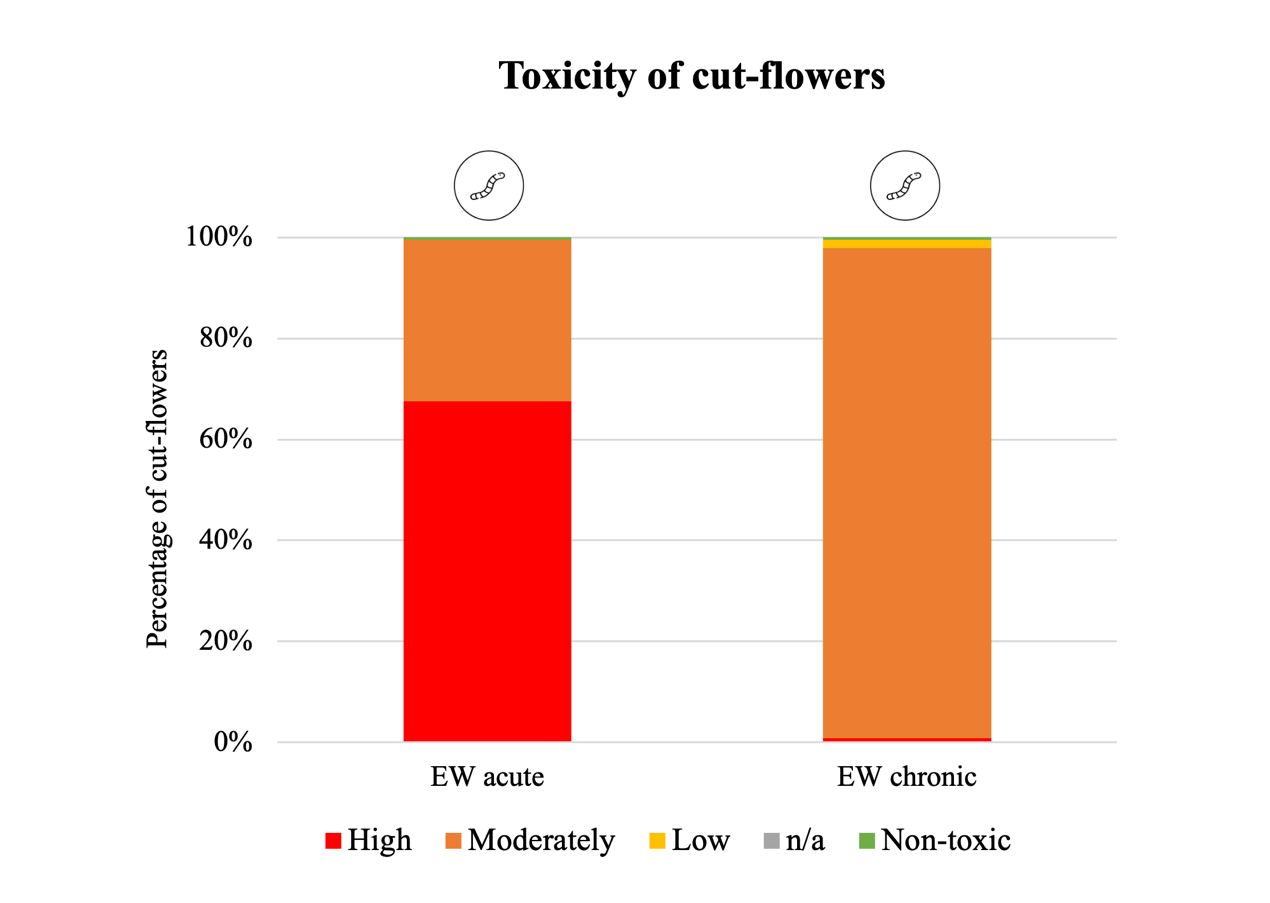


**Fig. S5.** Acute and chronic toxicity of cut-flowers (n= 237) for earth worms (EW). If several AIs were detected on a plant, the one with the highest toxicity was used for classifying the toxicity rating.


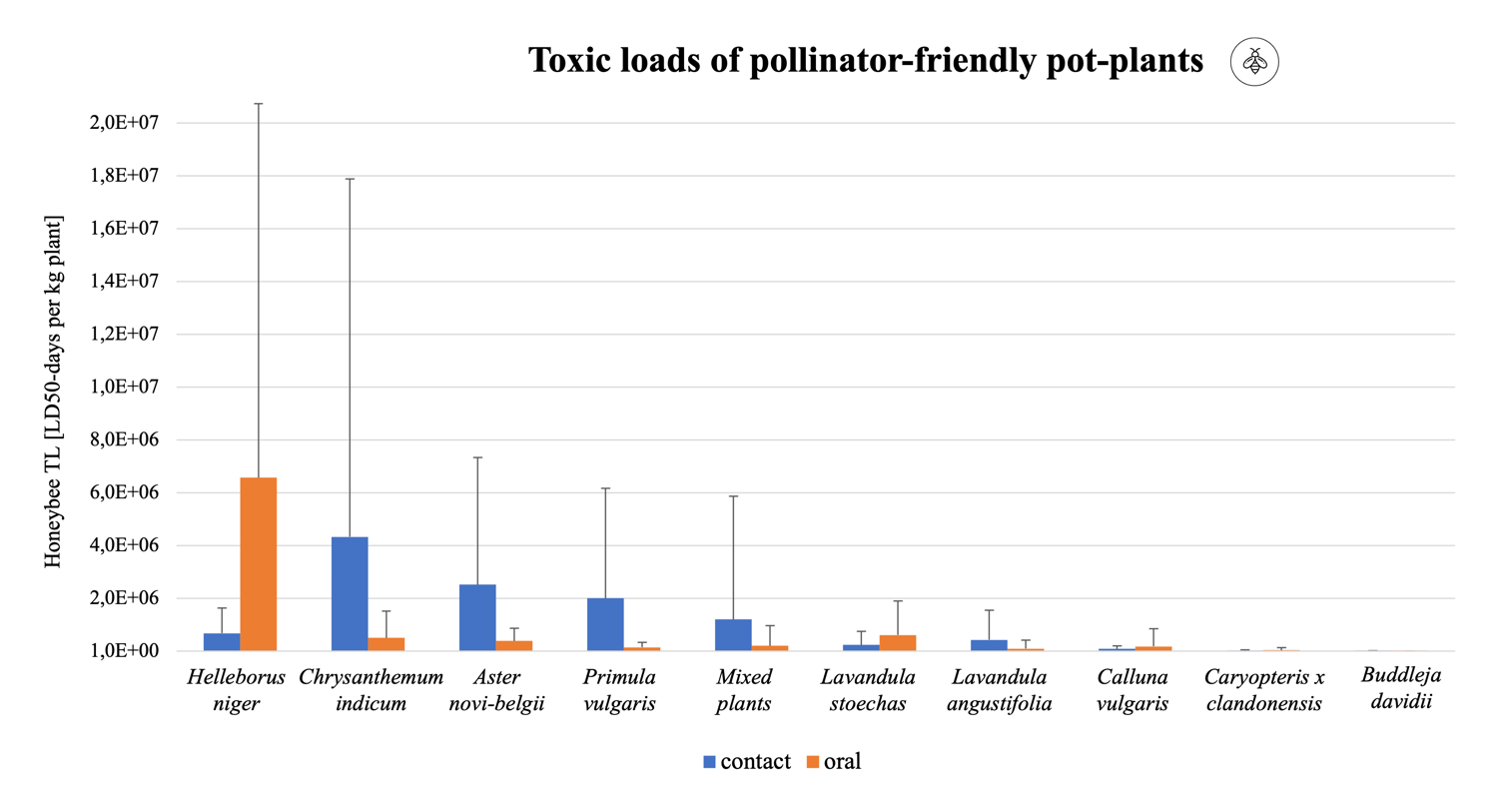


**Fig. S6.** Average TL (+SD) per plant for honeybees (contact/oral) calculated for the 10 pollinator-friendly pot-plant species featured most frequently in the dataset: *Helleborus niger* (n=9), *Chrysanthemum indicum* (n=18), *Aster novi-belgii* (n=8), *Primula vulgaris* (n=16), Mixed plants (n=15), *Lavandula stoechas* (n=12), *Lavandula angustifolia* (n= 45), *Calluna vulgaris* (n=17), *Caryopteris x clandonensis (*n=10), *Buddleja davidii* (n=6). They account for 51% of all pollinator-friendly pot-plants featured in the dataset. Standard deviation for *Helleborus niger* (oral) and *Chrysanthemum indicum* (contact) was not displayed here in detail due to high values and accounted for 1.4*10^7^ (oral) and 1.35*10^7^ (contact) respectively.


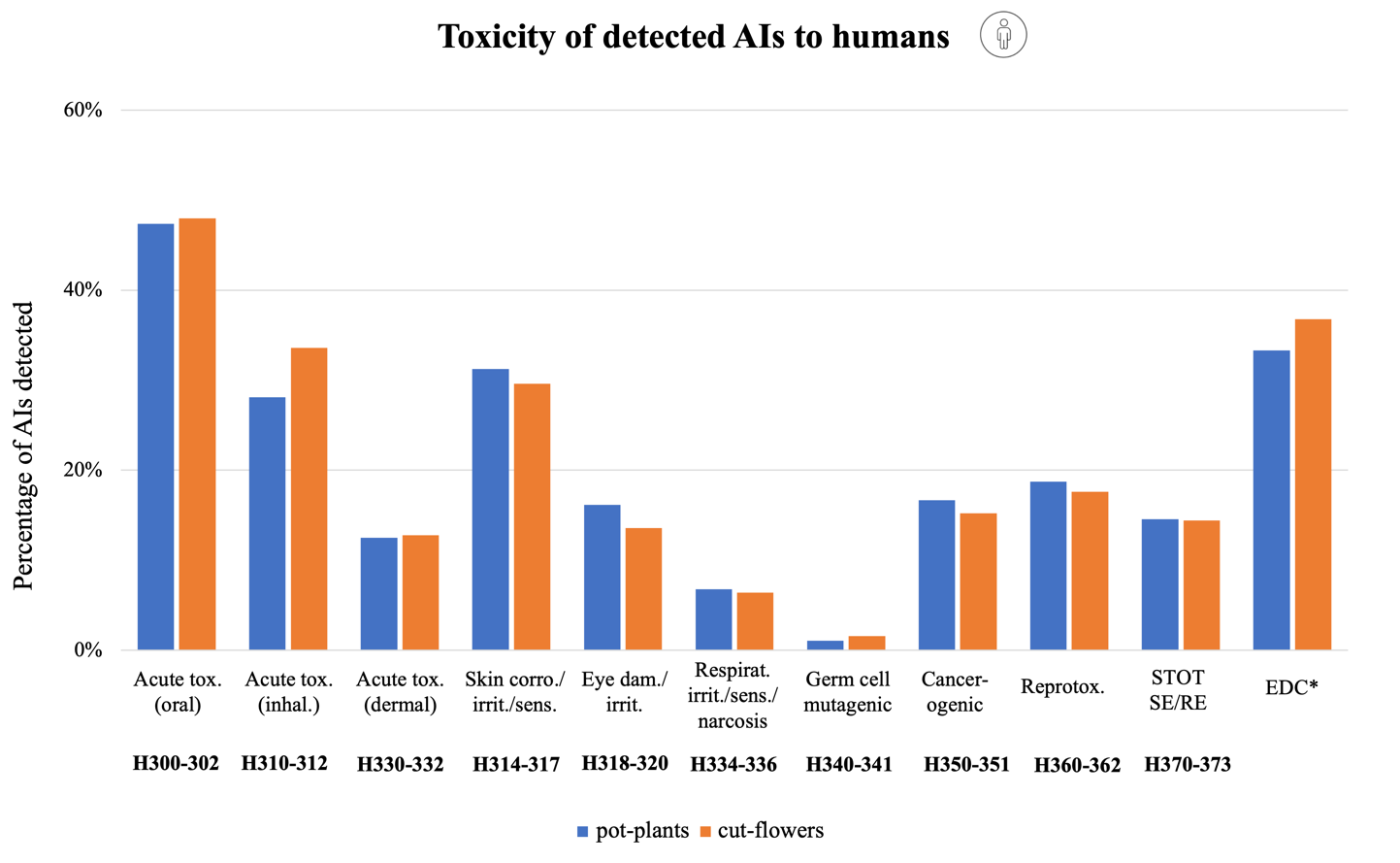


**Fig. S7.** Percentage of AIs detected on pot-plants (n=192) and cut-flowers (n=125) with human toxicological properties according to GHS hazard statements based on the EU regulation (EC) 1272/2008. Endocrine disrupting chemicals (EDC) were assessed based on PPDB.


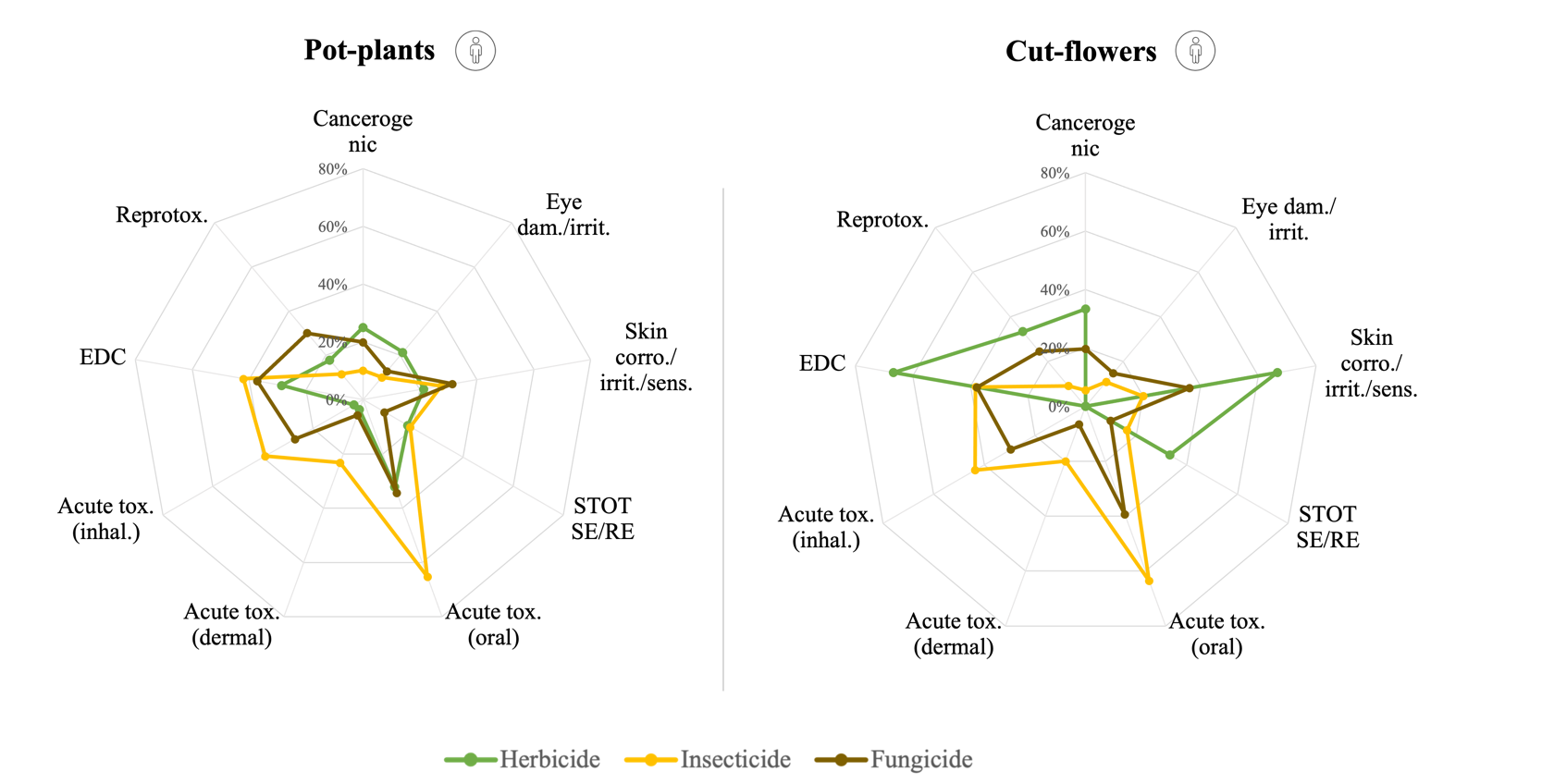


**Fig. S8.** Overview of human toxicological properties of pesticide types detected in 1,000 pot-plants (pp) and 237 cut-flowers (cf): herbicides (n_pp_=28, n_cf_=3), insecticides (n_pp_=69; n_cf_=55), and fungicides (n_pp_=70, n_cf_=61). Germ cell mutagenicity and respirational irritation/sensitisation/narcosis is not displayed in this diagram since they only apply to a small number of AIs. EDC…endocrine disrupting chemical, STOT SE/RE… specific target organ toxicity (single/repeated exposure), tox…toxicity, inhal…inhalational, dam…damaging, irrit…irritation, corro…corrosion, sens…sensitisation, reprotox…reproductive toxicity.
