# Supplementary material for: Ornamental plants as vectors of pesticide exposure and potential threat to biodiversity and human health
Source: Environ Sci Pollut Res Int. 2024 Jul 24;31(36):49079–99. doi: 10.1007/s11356-024-34363-x (PMC11310276; doi:10.1007/s11356-024-34363-x)
Supplement: Supplementary file 4 — Supplementary file4 (DOCX 87 KB) [file 11356_2024_34363_MOESM4_ESM.docx]

**Supplementary Tables S3-S11**

Manuscript Chwoyka et al: Ornamental plants as vectors of pesticide exposure and potential threat to biodiversity and human health.

**Table S3** Classification and interpretation of ecotoxicological thresholds (LD_50_, LC_50_, NOEC, NOEL and NOAEL values) for different species (Lewis et al., 2016).

| **Species group** | **Toxicity categories** | | |
| --- | --- | --- | --- |
|  | **High toxicity** | **Moderate toxicity** | **Low toxicity** |
| Mammals - LD_50_ acute oral [mg kg^-1^] | <100 | 100-2,000 | >2,000 |
| Mammals - NOAEL chronic [mg kg^-1^ d^-1^] | <10 | 10-200 | >200 |
| Birds - LD_50_ acute [mg kg^-1^] | <100 | 100-2,000 | >2,000 |
| Birds - NOEL chronic [mg kg^-1^ d^-1^] | <10 | 10-200 | >200 |
| Earthworms - LC_50_ acute [mg kg^-1^] | <10 | 10-1,000 | >1,000 |
| Earthworms - NOEC chronic [mg kg^-1^] | <0.1 | 0.1-100 | >100 |
| Honeybees - LD_50_ acute contact & oral  [μg bee^-1^] | <1 | 1-100 | >100 |
| Honeybees - LDD_50_ chronic | No interpretation | | |

**Table S4** Classification and interpretation of typical and field DT50 values (Kerle et al., 2007; Lewis et al., 2016)**.**

|  | **Very persistent** | **Persistent** | **Moderately persistent** | **Non-persistent** |
| --- | --- | --- | --- | --- |
| DT_50_ [days] | >365 | 100-365 | 30-99 | <30 |

**Table S5** Sub-classification of the GHS hazard statements based on the EU regulation (EC) 1272/2008 (United Nations, 2021).

| **Sub-class** | **Code** | **Hazard class and category** | **Hazard statement** |
| --- | --- | --- | --- |
| Acute toxicity  (oral) | H300 | Acute toxicity (oral), H-Cat* 1, 2 | Fatal if swallowed. |
|  | H301 | Acute toxicity (oral), H-Cat 3 | Toxic if swallowed. |
|  | H302 | Acute toxicity (oral), H-Cat 4 | Harmful if swallowed. |
| Acute toxicity (dermal) | H310 | Acute toxicity (dermal), H-Cat 1, 2 | Fatal in contact with skin. |
|  | H311 | Acute toxicity (dermal), H-Cat 3 | Toxic in contact with skin. |
|  | H312 | acute toxicity (dermal), H-Cat 4 | Harmful in contact with skin. |
| Skin corrosion/irritation/ sensitisation | H314 | Skin corrosion/irritation, H-Cat 1A, 1B, 1C | Causes severe skin burns and eye damage. |
|  | H315 | Skin corrosion/irritation, H-Cat 2 | Causes skin irritation. |
|  | H317 | Sensitisation — Skin, H-Cat 1 | May cause an allergic skin reaction. |
| Eye damage/ irritation | H318 | Serious eye damage/eye irritation, H-Cat 1 | Causes serious eye damage. |
|  | H319 | Serious eye damage/eye irritation, H-Cat 2A | Causes serious eye irritation. |
|  | H320 | Serious eye damage/eye irritation, H-Cat 2B | Causes eye irritation |
| Acute toxicity  (inhal.) | H330 | Acute toxicity (inhal.), H-Cat 1, 2 | Fatal if inhaled. |
|  | H331 | Acute toxicity (inhal.), H-Cat 3 | Toxic if inhaled. |
|  | H332 | Acute toxicity (inhal.), H-Cat 4 | Harmful if inhaled. |
| Respirational irritation/sensitisation/narcosis | H334 | Sensitisation — Respirat., H-Cat 1 | May cause allergy or asthma symptoms or breathing difficulties if inhaled. |
|  | H335 | Specific target organ toxicity — Single exposure, H-Cat 3, Respiratory tract irritation | May cause respiratory irritation. |
|  | H336 | Specific target organ toxicity — Single exposure, H-Cat 3, Narcosis | May cause drowsiness or dizziness. |
| Germ cell mutagenicity | H340 | Germ cell mutagenicity, H-Cat 1A, 1B | May cause genetic defects. |
|  | H341 | Germ cell mutagenicity, H-Cat 2 | Suspected of causing genetic defects. |
| Cancerogenic | H350 | Carcinogenicity, H-Cat 1A, 1B | May cause cancer. |
|  | H351 | Carcinogenicity, H-Cat 2 | Suspected of causing cancer. |
| Reproductive  toxicity | H360 | Reproductive toxicity, H-Cat 1A, 1B | May damage fertility or the unborn child. |
|  | H361 | Reproductive toxicity, H-Cat 2 | Suspected of damaging fertility or the unborn child. |
|  | H362 | Reproductive toxicity, Additional category, Effects on or via lactation | May cause harm to breast-fed children. |
| Specific target organ toxicity Single/Repeated exposure  (STOT SE/RE) | H370 | Specific target organ toxicity — single exposure, H-Cat 1 | Causes damage to organs. |
|  | H371 | Specific target organ toxicity — Single exposure, H-Cat 2 | May cause damage to organs. |
|  | H372 | Specific target organ toxicity — Repeated exposure, H-Cat 1 | Causes damage to organs through prolonged or repeated exposure. |
|  | H373 | Specific target organ toxicity — Repeated exposure, H-Cat 2 | May cause damage to organs through prolonged or repeated exposure. |

*H-cat = Hazard category

**Table S6** Top-5 pesticides with the highest total pesticide load for honeybees, birds, earthworms and rats detected on 1,000 pot-plants and 237 cut-flowers.

| **Pesticide** | **Pesticide type** | **Total pesticide load  [mg kg^-1^]** | **Frequency of detection across years** |
| --- | --- | --- | --- |
| **POT-PLANTS** |  |  |  |
| Chlormequat | plant growth regulator | 750 | 72 |
| 1-Naphthylacetamide | plant growth regulator | 649 | 2 |
| Folpet | Insecticide | 160 | 15 |
| Fenhexamid | fungicide | 157 | 103 |
| Paclobutrazol | plant growth regulator | 155 | 181 |
|  |  |  |  |
| **CUT-FLOWERS** |  |  |  |
| Clofentezine | acaricide | 190 | 54 |
| Propamocarb | fungicide | 94 | 116 |
| Iprodione | fungicide | 76 | 117 |
| BAC | fungicide | 53 | 4 |
| Dodemorph | fungicide | 50 | 100 |

**Table S7** Top-10 insecticides, fungicides, and herbicides detected on 1,000 pot-plants ranked by frequency of detection between 2013 and 2021. The pesticides’ EU permission as of 2022 was evaluated based on the EC Regulation 1107/2009 status stated by the PPDB. The half-life is based on DT50 field values sourced from the PPDB (Lewis et al., 2016).

| **Pesticide** | **EU perm.** | **Freq.  of  detection** | **% Freq.  of detection** | **Concentration [mg kg^-1^]**** | | | | | | **Half-life [days]** |
| --- | --- | --- | --- | --- | --- | --- | --- | --- | --- | --- |
|  |  |  |  | **min** | **max** | **mean±SD** | | | **median** |  |
| **INSECTICIDES** |  |  |  |  |  |  |  |  |  |  |
| Flonicamid | ✓ | 487 | 49% | 0.004 | 110 | 2.303 | ± | 6.723 | 0.350 | 3.1 |
| Spinosad* | ✓ | 118 | 12% | 0.01 | 13.6 | 0.985 | ± | 2.198 | 0.094 | 14 |
| Lambda-cyhalothrin* | ✓ | 100 | 10% | 0.01 | 14 | 0.595 | ± | 1.716 | 0.195 | 26.9 |
| Spirotetramat | ✓ | 93 | 9% | 0.01 | 8.1 | 0.709 | ± | 1.475 | 0.14 | 0.7 |
| Pirimicarb* | ✓ | 88 | 9% | 0.01 | 18.7 | 0.829 | ± | 2.194 | 0.168 | 9 |
| Pymetrozine* | x | 81 | 8% | 0.004 | 22 | 2.569 | ± | 5.161 | 0.27 | 22.6 |
| Acetamiprid | ✓ | 75 | 8% | 0.003 | 4.7 | 0.634 | ± | 1.092 | 0.16 | 3 |
| Thiacloprid* | x | 72 | 7% | 0.006 | 9.4 | 0.709 | ± | 1.482 | 0.14 | 8.1 |
| Indoxacarb* | ✓ | 70 | 7% | 0.008 | 6.2 | 0.435 | ± | 0.906 | 0.125 | 5.97 |
| Azadirachtin | ✓ | 63 | 6% | 0.01 | 2.8 | 0.288 | ± | 0.492 | 0.081 | 5 |
|  |  |  |  |  |  |  |  |  |  |  |
| **FUNGICIDES** |  |  |  |  |  |  |  |  |  |  |
| Boscalid | ✓ | 350 | 35% | 0.005 | 22 | 1.452 | ± | 3.329 | 0.098 | 254 |
| Propiconazole* | x | 247 | 25% | 0.007 | 21 | 1.247 | ± | 2.839 | 0.140 | 35.2 |
| Fluopyram | ✓ | 233 | 23% | 0.004 | 70 | 0.915 | ± | 4.737 | 0.077 | 118.8 |
| Pyraclostrobin | ✓ | 175 | 18% | 0.003 | 6.7 | 0.349 | ± | 0.759 | 0.087 | 33.3 |
| Azoxystrobin | ✓ | 167 | 17% | 0.004 | 16 | 1.140 | ± | 2.679 | 0.125 | 180.7 |
| Propamocarb | ✓ | 157 | 16% | 0.004 | 22.2 | 1.041 | ± | 2.788 | 0.115 | 14 |
| Difenoconazole | ✓ | 153 | 15% | 0.001 | 17 | 0.638 | ± | 1.770 | 0.080 | 91.8 |
| Cyprodinil | ✓ | 144 | 14% | 0.006 | 19 | 0.812 | ± | 2.384 | 0.069 | 45 |
| Iprodione* | x | 128 | 13% | 0.01 | 39.9 | 2.293 | ± | 5.374 | 0.190 | 11.7 |
| Carbendazim* | x | 111 | 11% | 0.005 | 5.6 | 0.398 | ± | 0.909 | 0.080 | 22 |
|  |  |  |  |  |  |  |  |  |  |  |
| **HERBICIDES** |  |  |  |  |  |  |  |  |  |  |
| Oxadiazon* | x | 15 | 2% | 0.01 | 0.09 | 0.035 | ± | 0.027 | 0.024 | 165 |
| Pendimethalin* | ✓ | 15 | 2% | 0.01 | 0.29 | 0.052 | ± | 0.079 | 0.016 | 100.6 |
| Linuron* | x | 14 | 1% | 0.01 | 0.061 | 0.030 | ± | 0.017 | 0.029 | 48 |
| Haloxyfop | x | 13 | 1% | 0.021 | 4.6 | 1.016 | ± | 1.434 | 0.260 | 9 |
| Isoxaben | ✓ | 13 | 1% | 0.007 | 1.2 | 0.184 | ± | 0.386 | 0.024 | 123 |
| Propyzamid | ✓ | 10 | 1% | 0.011 | 2.3 | 0.285 | ± | 0.709 | 0.068 | 233 |
| Quinoclamine* | x | 8 | 1% | 0.025 | 45 | 6.419 | ± | 15.66 | 0.285 | 22 |
| Fluazifop-p-butyl | ✓ | 6 | 1% | 0.019 | 0.049 | 0.033 | ± | 0.012 | 0.034 | 8.2 |
| Terbuthylazine | ✓ | 6 | 1% | 0.025 | 0.051 | 0.038 | ± | 0.012 | 0.037 | 21.8 |
| Metribuzin* | ✓ | 5 | 1% | 0.01 | 0.032 | 0.016 | ± | 0.009 | 0.012 | 19 |

*Known as “highly hazardous pesticide” (PAN, 2021)

**Minimum values, mean values, standard deviation, and median were calculated by excluding zeros.

**Table S8** Top-10 insecticides, fungicides, and herbicides on 237 cut-flowers ranked by frequency of detection between 2011 and 2021. The pesticides’ EU permission as of 2022 was evaluated based on the EC Regulation 1107/2009 status stated by the PPDB. The half-life is based on DT50 field values sourced from the PPDB (Lewis et al., 2016).

| **Pesticide** | **EU perm.** | **Freq.  of  detection** | **% Freq.  of  detection** | **Concentration [mg kg^-1^]**** | | | | | | **Half-life [days]** |
| --- | --- | --- | --- | --- | --- | --- | --- | --- | --- | --- |
|  |  |  |  | **min** | **max** | **mean±SD** | | | **median** |  |
| **INSECTICIDES** |  |  |  |  |  |  |  |  |  |  |
| Acetamiprid | ✓ | 136 | 57% | 0.01 | 3.9 | 0.589 | ± | 0.797 | 0.230 | 3 |
| Lufenuron* | ✓ | 66 | 28% | 0.011 | 3.373 | 0.537 | ± | 0.660 | 0.245 | 256 |
| Imidacloprid* | x | 65 | 27% | 0.009 | 6.2 | 0.528 | ± | 1.040 | 0.140 | 174 |
| Acephate* | x | 43 | 18% | 0.011 | 47 | 5.123 | ± | 10.051 | 1.200 | 3 |
| Indoxacarb* | ✓ | 41 | 17% | 0.01 | 2.8 | 0.383 | ± | 0.634 | 0.170 | 5.97 |
| Methamidophos* | x | 40 | 17% | 0.011 | 10.8 | 1.194 | ± | 2.036 | 0.440 | 4 |
| Flubendiamide* | ✓ | 34 | 14% | 0.01 | 5.2 | 0.814 | ± | 0.999 | 0.565 | 500 |
| Spinosad* | ✓ | 30 | 13% | 0.013 | 5.9 | 0.904 | ± | 1.399 | 0.425 | 14 |
| Buprofezin | ✓ | 30 | 13% | 0.005 | 24.6 | 2.104 | ± | 5.507 | 0.036 | 45.6 |
| Thiamethoxam* | x | 25 | 11% | 0.01 | 3.78 | 0.328 | ± | 0.790 | 0.033 | 39 |
|  |  |  |  |  |  |  |  |  |  |  |
| **FUNGICIDES** |  |  |  |  |  |  |  |  |  |  |
| Spiroxamine | ✓ | 211 | 89% | 0.005 | 27 | 4.148 | ± | 6.171 | 2.000 | 52.4 |
| Iprodione* | x | 117 | 49% | 0.01 | 76.2 | 5.000 | ± | 9.886 | 1.100 | 11.7 |
| Propamocarb | ✓ | 116 | 49% | 0.01 | 94.33 | 4.241 | ± | 10.816 | 0.460 | 14 |
| Pyrimethanil | ✓ | 116 | 49% | 0.01 | 19.32 | 1.854 | ± | 3.448 | 0.258 | 31.4 |
| Boscalid | ✓ | 108 | 46% | 0.01 | 27.3 | 2.222 | ± | 4.321 | 0.349 | 254 |
| Dodemorph | ✓ | 100 | 42% | 0.011 | 49.5 | 4.431 | ± | 9.338 | 0.590 | 41 |
| Cyprodinil | ✓ | 85 | 36% | 0.01 | 6.9 | 0.657 | ± | 1.309 | 0.068 | 45 |
| Fenhexamid* | ✓ | 71 | 30% | 0.013 | 12.6 | 1.940 | ± | 2.654 | 0.720 | 0.43 |
| Ethirimol* | x | 63 | 27% | 0.007 | 1.179 | 0.155 | ± | 0.230 | 0.061 | 20 |
| Dimethomorph | ✓ | 55 | 23% | 0.01 | 17 | 1.664 | ± | 3.262 | 0.216 | 44 |
|  |  |  |  |  |  |  |  |  |  |  |
| **HERBICIDES**** |  |  |  |  |  |  |  |  |  |  |
| Atrazine | x | 1 | 0.4% | 0.010 | 0.010 | - | ± | - | - | 29 |
| Fluazifop-p-butyl | ✓ | 1 | 0.4% | 0.140 | 0.140 | - | ± | - | - | 8.2 |
| Trifluralin* | x | 1 | 0.4% | 0.020 | 0.020 | - | ± | - | - | 170 |

*Known as “highly hazardous pesticide” (PAN, 2021).

**Minimum values, mean values, standard deviation and median were calculated by excluding zeros.
*** Only 3 herbicides were detected on cut flowers. Each of them only occurred one time, thus no mean, standard deviation, and median were calculated.

**Table S9** Top-10 AIs detected on E. pulcherrima (n=77) and their human toxicological properties. None of the AIs were eye damaging/irritating, cause respirational irritation/ sensitisation/narcosis or was mutagenic to germ cells; therefore, these properties are not shown in this table. Check marks (✓) mean yes/applicable; x means no/not applicable.

| **Pesticide** | **Freq. of detection** | **EU perm.** | **Half-life [days]** | **Humantox** | | | | | | | |
| --- | --- | --- | --- | --- | --- | --- | --- | --- | --- | --- | --- |
|  |  |  |  | **Acute tox. (oral)** | **Acute tox. (dermal)** | **Acute tox. (inhal.)** | **Skin corro./ irrit./sens.** | **Cancero-genic** | **Repro-tox.** | **STOT SE/RE** | **EDC** |
| Chlormequat | 51 | ✓ | 27.4 | ✓ | ✓ | x | x | x | x | x | x |
| Flonicamid | 49 | ✓ | 3.1 | ✓ | x | x | x | x | x | x | x |
| Boscalid | 20 | ✓ | 254 | x | x | x | x | x | x | x | x |
| Fluopyram | 18 | ✓ | 118.8 | x | x | x | x | x | x | x | x |
| Propamocarb | 16 | ✓ | 14 | ✓ | x | x | x | x | x | x | ✓ |
| Pymetrozine | 15 | x | 22.6 | x | x | x | x | ✓ | x | x | x |
| Abamectin | 13 | ✓ | 1 | ✓ | x | ✓ | x | x | ✓ | ✓ | x |
| Propiconazole | 12 | x | 35.2 | ✓ | x | x | ✓ | x | ✓ | x | ✓ |
| Pyraclostrobin | 12 | ✓ | 33.3 | x | x | ✓ | ✓ | x | x | x | x |
| Spinosad | 11 | ✓ | 14 | x | x | x | x | x | x | x | x |

**Table S10** Overview of the country of origin, the average number of AIs per plant (± standard deviation, as well as the average pesticide load of each plant (± standard deviation) for pot-plants and cut-flowers. Countries were ranged by the respective number of plants originating from it.

|  | **no. of plants analysed** | **no. of  AIs/plant** | | | **pesticide load/plant  [mg kg^-1^]** | | |  |
| --- | --- | --- | --- | --- | --- | --- | --- | --- |
|  |  | mean | ± | SD | mean | ± | SD | |
| **POT-PLANTS** |  |  |  |  |  |  |  | |
| Unknown | 455 | 5.1 | ± | 3.8 | 11.8 | ± | 52.3 | |
| Germany | 333 | 6.9 | ± | 4.2 | 21.7 | ± | 55.2 | |
| Netherlands | 95 | 6.3 | ± | 4.1 | 11.6 | ± | 27.8 | |
| Austria | 62 | 3.9 | ± | 3.8 | 2.7 | ± | 6.5 | |
| Italy | 28 | 6.2 | ± | 3.7 | 28.7 | ± | 123.0 | |
| Denmark | 14 | 4.6 | ± | 2.0 | 9.3 | ± | 9.8 | |
| Spain | 5 | 4.2 | ± | 2.6 | 2.7 | ± | 4.9 | |
| Portugal | 4 | 5.5 | ± | 1.7 | 6.8 | ± | 12.8 | |
| Belgium | 1 | 15.0 | ± | 0.0 | 3.8 | ± | 0.0 | |
| France | 1 | 7.0 | ± | 0.0 | 1.9 | ± | 0.0 | |
| Poland | 1 | 2.0 | ± | 0.0 | 0.1 | ± | 0.0 | |
| Vietnam | 1 | 8.0 | ± | 0.0 | 39.7 | ± | 0.0 | |
|  |  |  |  |  |  |  |  | |
| **CUT-FLOWERS** |  |  |  |  |  |  |  | |
| Kenya | 120 | 14.6 | ± | 5.6 | 30.1 | ± | 33.4 | |
| Tanzania | 97 | 6.8 | ± | 3.4 | 11.9 | ± | 11.9 | |
| Unknown | 11 | 10.2 | ± | 6.2 | 27.7 | ± | 61.6 | |
| Netherlands | 3 | 4.3 | ± | 5.1 | 5.2 | ± | 8.8 | |
| Costa Rica | 2 | 6.5 | ± | 4.9 | 48.4 | ± | 23.1 | |
| Ecuador | 2 | 20.0 | ± | 4.2 | 26.1 | ± | 14.2 | |
| Germany | 1 | 16.0 | ± | 0.0 | 17.1 | ± | 0.0 | |
| Austria | 1 | 1.0 | ± | 0.0 | 0.01 | ± | 0.0 | |

**Table S11** Comparison of the results of this study with the results of other studies (Global 2000, 2021; Global 2000, 2022; Greenpeace, 2014; Lentola et al., 2017; Toumi et al., 2016a; Toumi et al., 2016b).

|  | **Sample size** | **Total AIs detected** | **Mean no. of AIs/ plant** | **Contam-inated plants** | **Plants with**  **> 1 AI** | **Plants with AIs not approved in EU** | | **AIs not approved in EU** | **Location of sampling** | **Year of analysis** |
| --- | --- | --- | --- | --- | --- | --- | --- | --- | --- | --- |
| **POT-PLANTS** |  |  |  |  |  |  | |  |  |  |
| This study | 1000 | 195 | 5,8 | 94% | 84% | 60% | | 37%* | AT | 2013-2021 |
| Lentola et al. | 29 | 13 | - | 94% | 79% | - | | - | UK | 2017 |
| Global 2000 (a) | 44 | 64 | 7,7 | 91% | 93% | 39% | | 22% | AT, GER | 2022 |
| Global 2000 (b) | 35 | 55 | 7 | 91% | 89% | 40% | | - | AT, GER | 2020 |
| Greenpeace | 86 | - | - | 98% | - | 14% | | - | EU | 2014 |
|  |  |  |  |  |  | |  |  |  |  |
| **CUT-FLOWERS** | | | | | | | | | | |
| This study | 237 | 126 | 11 | 99,6% | 98% | 85% | | 42% | AT | 2011-2021 |
| Toumi et al. (a) | 90 | 107 | 10 | 99% | - | - | | - | BE | 2016 |
| Toumi et al. (b) | 50 | 97 | 14 | 100% | - | - | | 20% | BE | 2016 |

*not approved as of 2022
